# Supplementary material for: Single-cell glycome and transcriptome profiling uncovers the glycan signature of each cell subpopulation of human iPSC-derived neurons
Source: Stem Cell Reports. 2025 Sep 4;20(10):102631. doi: 10.1016/j.stemcr.2025.102631 (PMC12790714; doi:10.1016/j.stemcr.2025.102631)
Supplement: Document S1. Figures S1–S5 and supplemental methods [file mmc1.pdf]

Stem Cell Reports, Volume *20*

Supplemental Information

Single-cell glycome and transcriptome profiling uncovers the glycan signature of each cell subpopulation of human iPSC-derived neurons

Haruki Odaka and Hiroaki Tateno

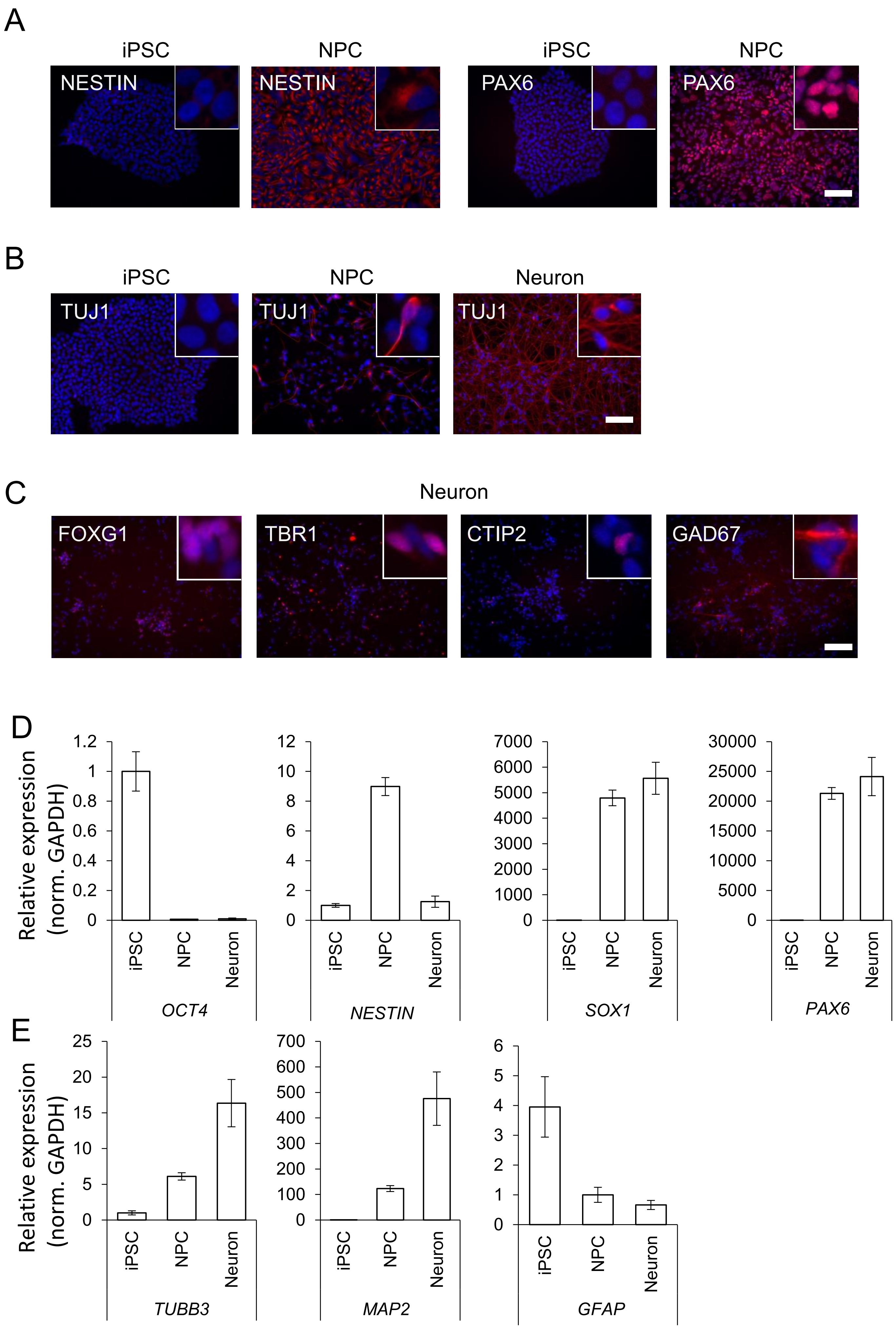

A
iPSC
NPC
iPSC
NPC
NESTIN
NESTIN
PAX6
PAX6
B
iPSC
NPC
Neuron
TUJ1
TUJ1
TUJ1
C
Neuron
FOXG1
TBR1
CTIP2
GAD67
D
Relative expression (norm. GAPDH)
OCT4
NESTIN
SOX1
PAX6
iPSC
NPC
Neuron
E
Relative expression (norm. GAPDH)
TUBB3
MAP2
GFAP
iPSC
NPC
Neuron

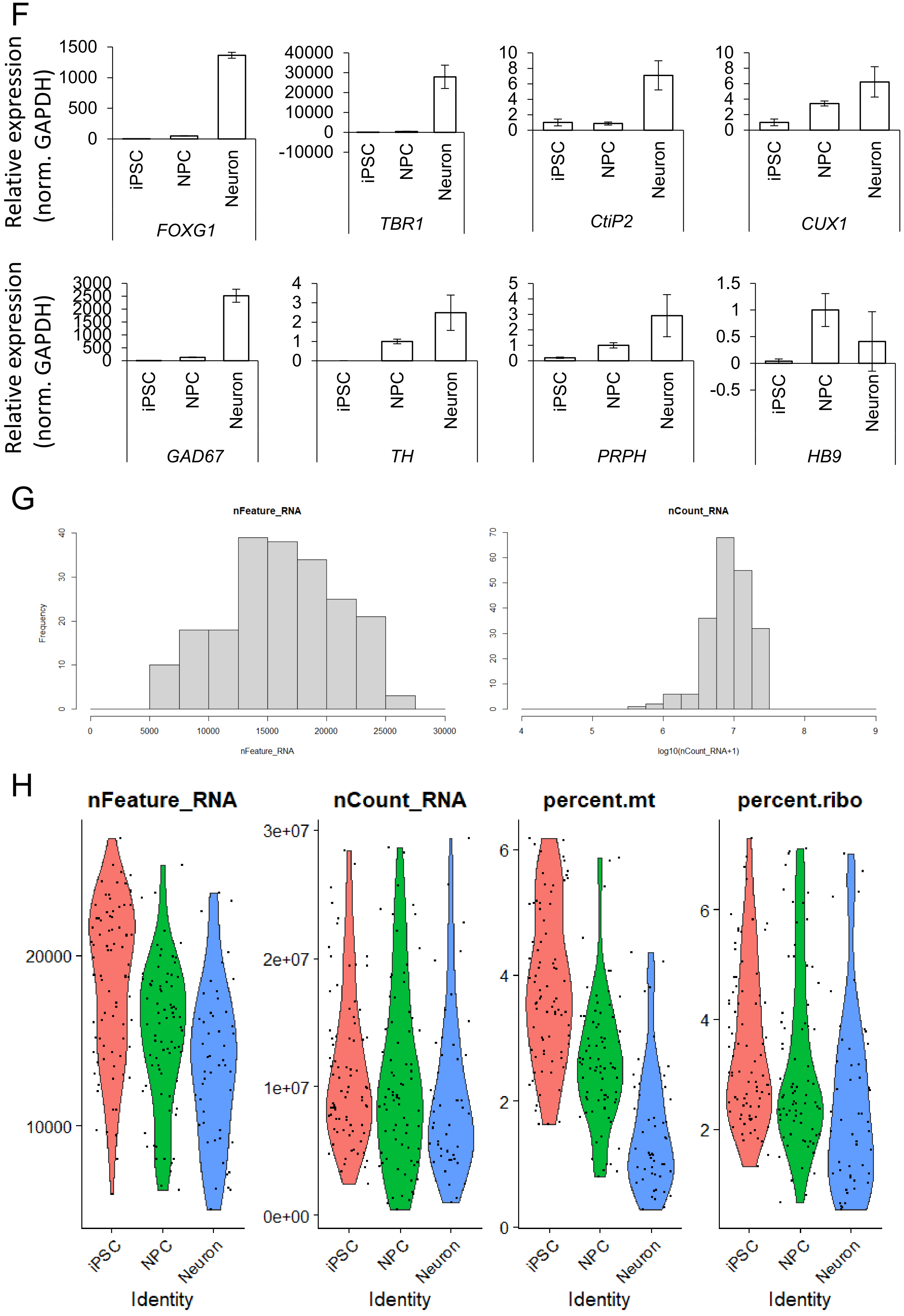
F
Relative expression (norm. GAPDH)
FOXG1
TBR1
CtiP2
CUX1
GAD67
TH
PRPH
HB9
iPSC
NPC
Neuron
G
nFeature_RNA
Frequency
nCount_RNA
log10(nCount_RNA+1)
H
nFeature_RNA
nCount_RNA
percent.mt
percent.ribo
Identity

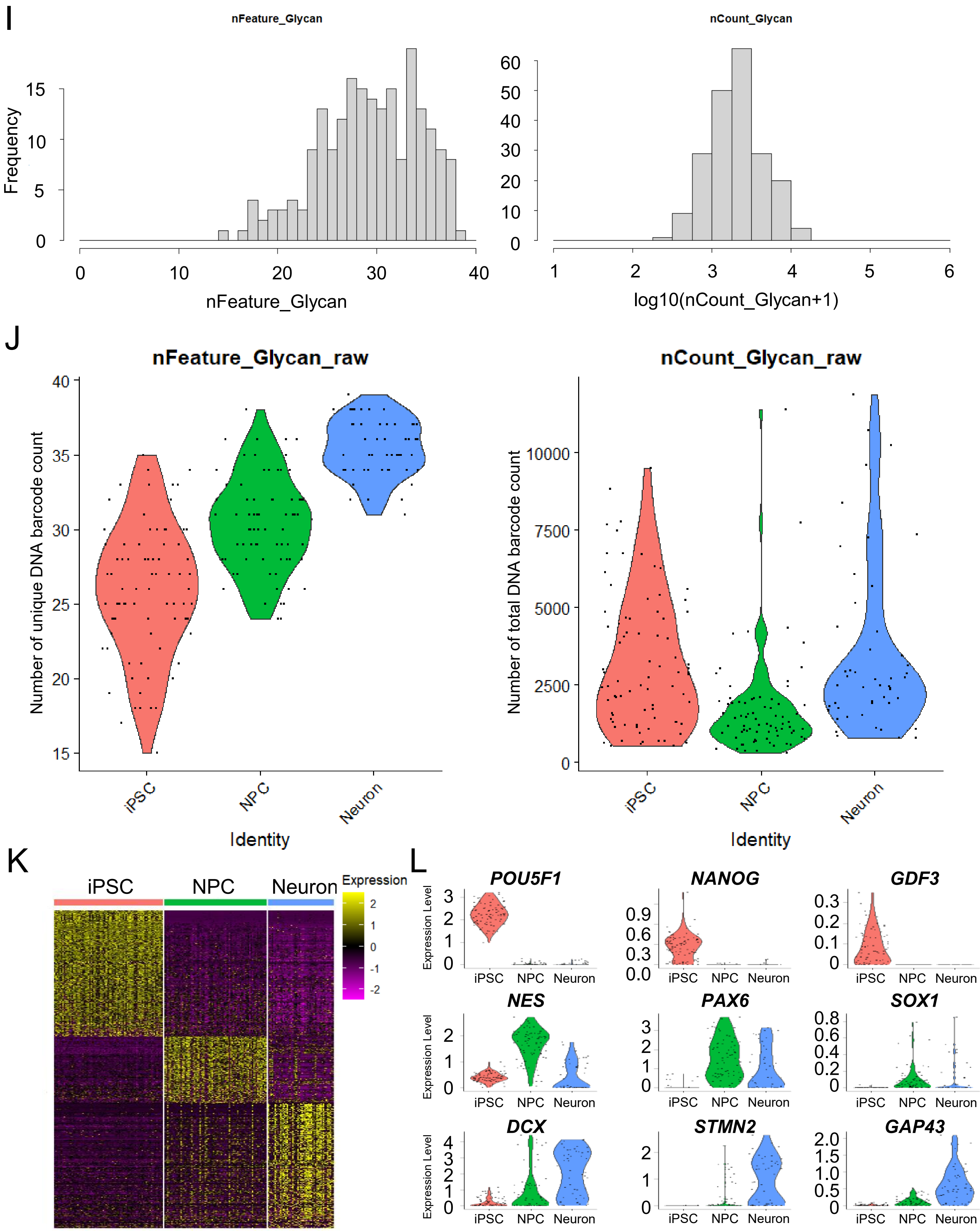
I
nFeature_Glycan
nCount_Glycan
Frequency
0
5
10
15
0
10
20
30
40
nFeature_Glycan
0
10
20
30
40
50
60
1
2
3
4
5
6
log10(nCount_Glycan+1)
J
nFeature_Glycan_raw
nCount_Glycan_raw
Number of unique DNA barcode count
15
20
25
30
35
40
Number of total DNA barcode count
0
2500
5000
7500
10000
iPSC
NPC
Neuron
Identity
iPSC
NPC
Neuron
Identity
K
iPSC
NPC
Neuron
Expression
2
1
0
-1
-2
L
POU5F1
NANOG
GDF3
NES
PAX6
SOX1
DCX
STMN2
GAP43
Expression Level
iPSC
NPC
Neuron

Fig. S1. scGR-seq analysis for iPSC, NPC, and neurons.
(A) Immunofluorescent staining of NPC markers (NESTIN, PAX6) in iPSCs and NPCs. (B) Immunofluorescent staining of a neuron marker (TUJ1) in iPSCs, NPCs and neurons. (C) Immunofluorescent staining of telencepharon (FOXG1), cortical deep-layer neuron (TBR1, CTIP2), and GABAergic neuron (GAD67) markers in neurons. Scale bar: 100 μm (D) Relative mRNA expression of a pluripotent stem cell marker (*OCT4*) and NPC markers (*NESTIN*, *SOX1*, *PAX6*) in iPSCs, NPCs, and neurons (n=3 , each from a separate well). Expression levels of each marker were normalized with *GAPDH* expression. (E) Relative mRNA expression of neuron markers (*TUBB3*, *MAP2*) and an astrocyte marker (*GFAP*) in iPSCs, NPCs, and neurons (n=3 , each from a separate well). (F) Relative mRNA expression of telencepharon (*FOXG1*), cortical deep-layer neuron (*TBR1*, *CTIP2*), cortical upper-layer neuron(*CUX1*), GABAergic neuron (*GAD67*), dopaminergic neuron (*TH*), peripheral neuron (*PRPH*), and motor neuron (*HB9*) markers in iPSCs, NPCs, and neurons (n=3 , each from a separate well). (G) Histogram of the unique genes detected in each cell and the total number of mapped read counts (n=206 cells). (H) Violin plots of the number of unique genes detected in each cell, the total number of mapped read counts, the percentage of mitochondrial genes, and the percentage of genes encoding ribosomal proteins in iPSC (n=81 cells), NPC (n=76 cells), and neurons (n=49 cells). (I) Histogram of the number of unique DNA barcodes (nFeature_Glycan) detected and the total DNA barcode counts (nCount_Glycan) (n=206 cells). (J) Violin plot of number of unique DNA and the total DNA barcode counts in iPSC (n=81 cells), NPC (n=76 cells), and neurons (n=49 cells). (K) Heatmap of differentially expressed genes (DEGs) in iPSC, NPC, and neurons. Criteria for DEG selection was set at log2(FoldChange) > 0.25 and Benjamini-Hochberg adjusted $p < 0.05$ (Mann-Whitney U test). All gene lists are shown in Supplementary Table 1. (L) Violin plots of canonical marker gene expressions of iPSCs (*POU5F1*, *NANOG*, *GDF3*), NPC (*NES*, *PAX6*, *SOX1*) and neurons (*DCX*, *STMN2*, *GAP43*) in each group. iPSC: n=81 cells, NPC: n=76 cells, neuron: n=49 cells.

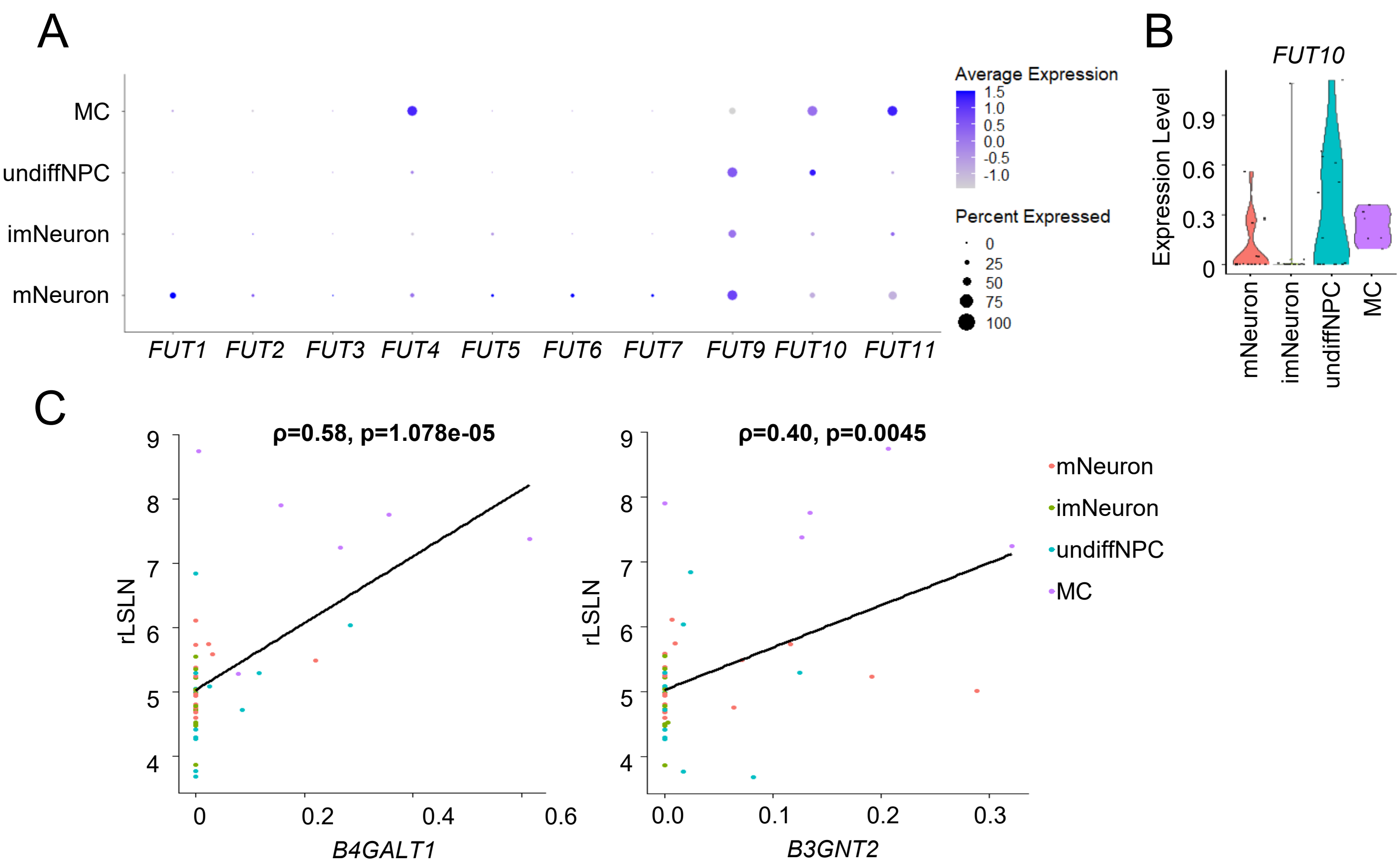

Fig. S2 Gene expression of glycosyltransferases in sub-population of neuron culture.

(A) Dotplots of fucosyltransferase-encoding genes expression in a subpopulation of neurons. (B) Violin plots of the mRNA expression of *FUT10*. (C) Scatter plots showing the relationship between rLSLN binding intensity and the expression levels of *B4GALT1* and *B3GNT2*. The black line represents the fitted regression line. ρ: Spearman's rank correlation coefficient; p: p-value from Spearman's rank correlation test.

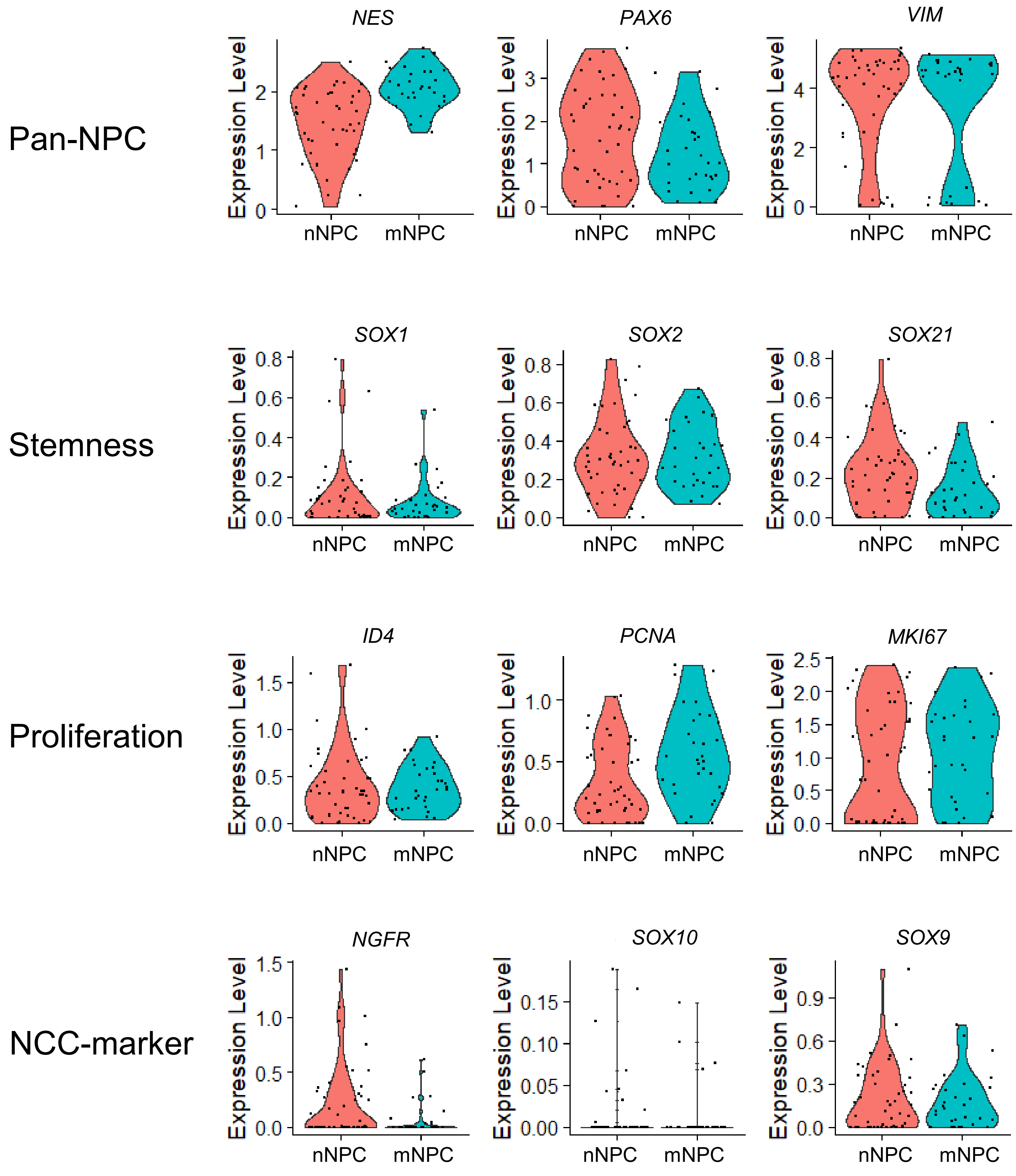

Fig. S3. Gene expression analysis in NPC subpopulations.
Violin plots of the gene expression of stemness markers (SOX1, SOX2 and SOX21), proliferation markers (ID4, PCNA, and MKI67), and neural crest cell markers (NGFR, SOX10, and SOX9) obtained by scRNA-seq analysis.

A

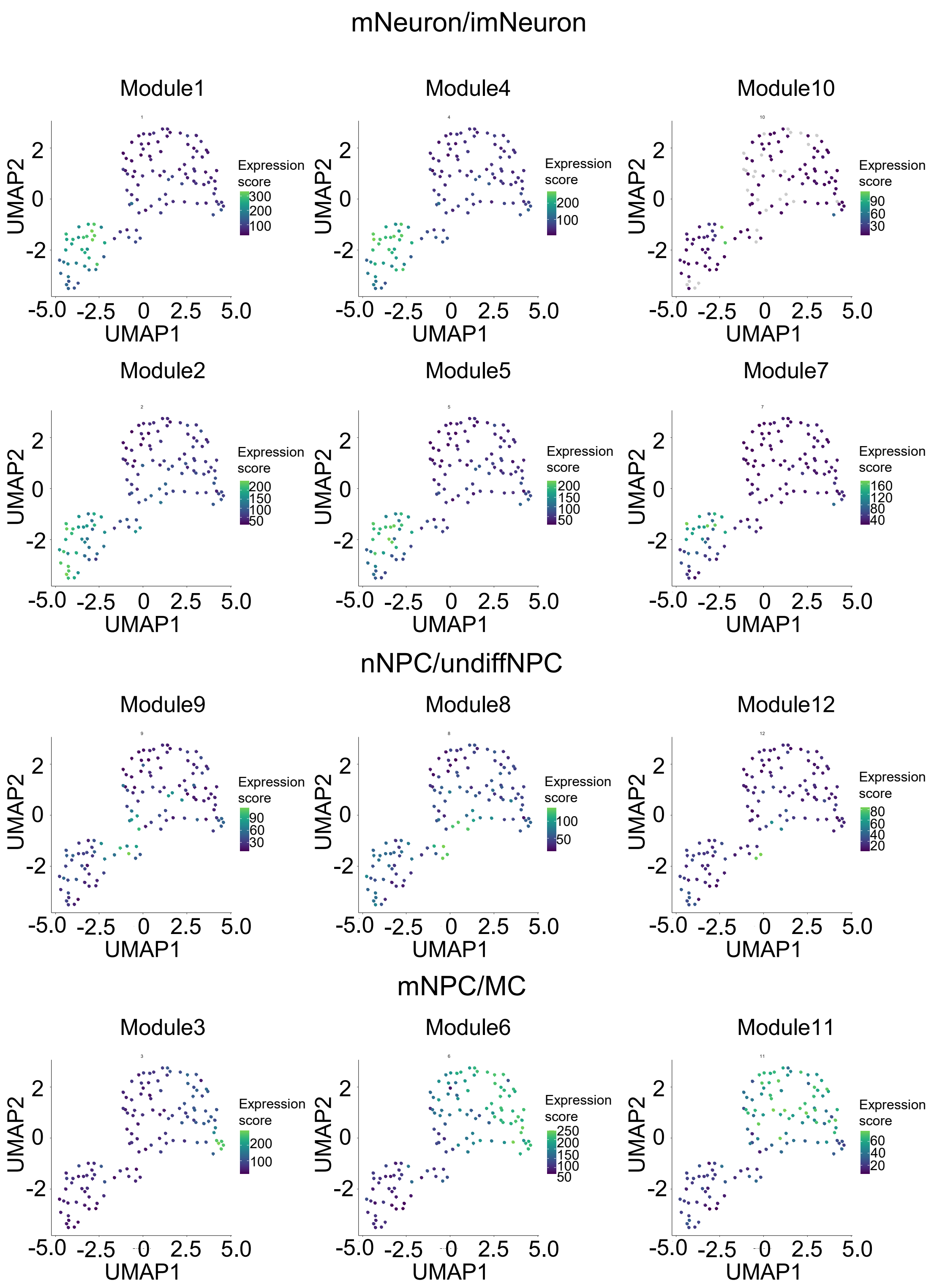
mNeuron/imNeuron
Module1
Module4
Module10
Module2
Module5
Module7
nNPC/undiffNPC
Module9
Module8
Module12
mNPC/MC
Module3
Module6
Module11
UMAP1
UMAP2
Expression score

B

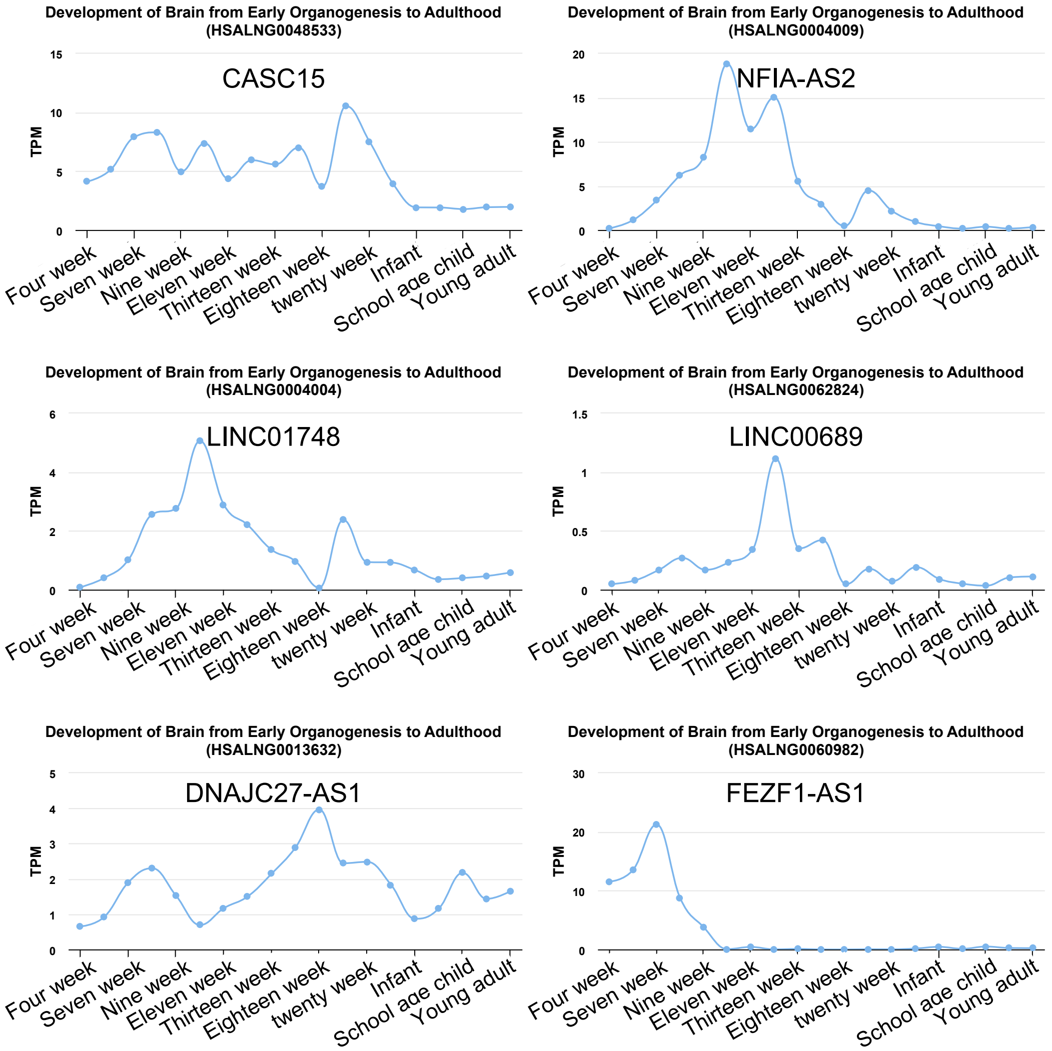
Development of Brain from Early Organogenesis to Adulthood
(HSALNG0048533)
CASC15
TPM
Development of Brain from Early Organogenesis to Adulthood
(HSALNG0004009)
NFIA-AS2
TPM
Development of Brain from Early Organogenesis to Adulthood
(HSALNG0004004)
LINC01748
TPM
Development of Brain from Early Organogenesis to Adulthood
(HSALNG0062824)
LINC00689
TPM
Development of Brain from Early Organogenesis to Adulthood
(HSALNG0013632)
DNAJC27-AS1
TPM
Development of Brain from Early Organogenesis to Adulthood
(HSALNG0060982)
FEZF1-AS1
TPM
Four week
Seven week
Nine week
Eleven week
Thirteen week
Eighteen week
twenty week
Infant
School age child
Young adult

C

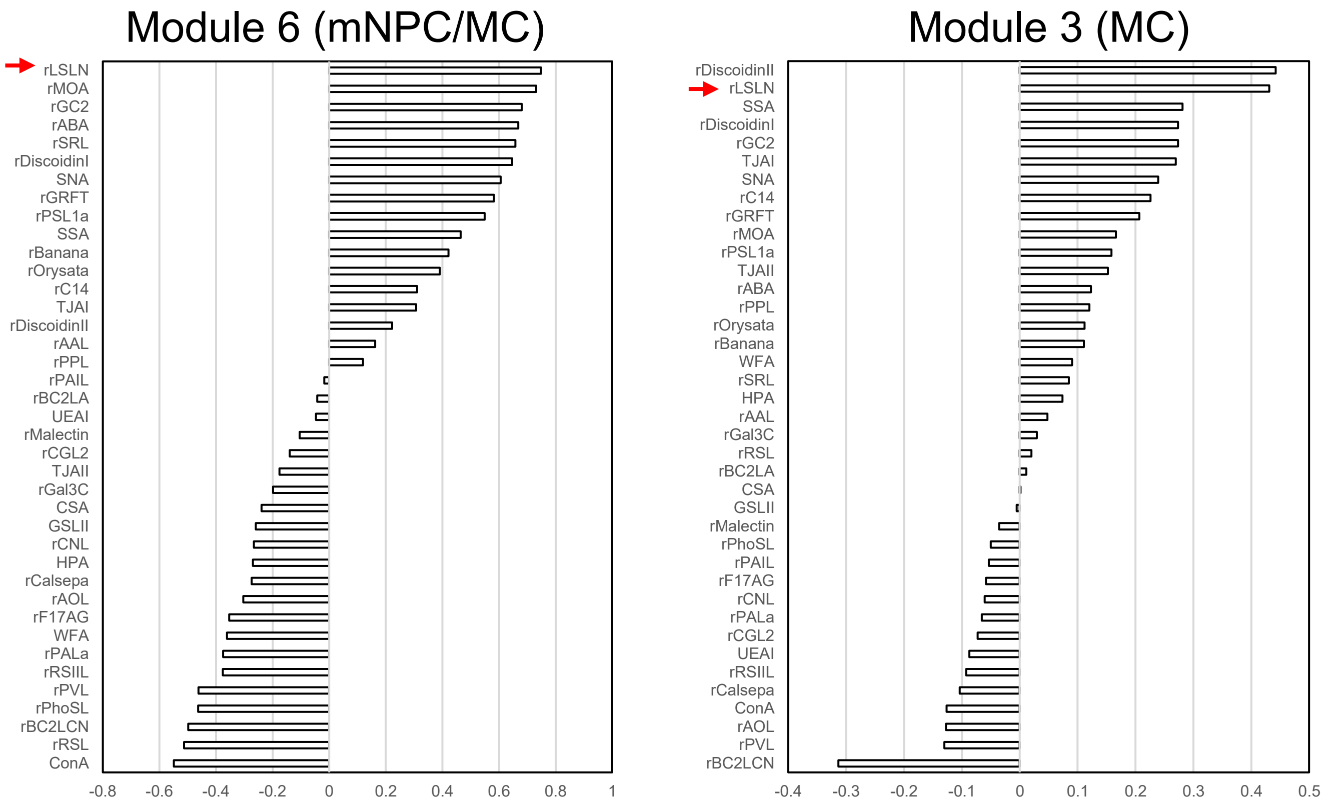

Module 6 (mNPC/MC)
rLSLN
rMOA
rGC2
rABA
rSRL
rDiscoidinI
SNA
rGRFT
rPSL1a
SSA
rBanana
rOrysata
rC14
TJAI
rDiscoidinII
rAAL
rPPL
rPAIL
rBC2LA
UEAI
rMalectin
rCGL2
TJAII
rGal3C
CSA
GSLII
rCNL
HPA
rCalsepa
rAOL
rF17AG
WFA
rPALa
rRSIIL
rPVL
rPhoSL
rBC2LCN
rRSL
ConA
-0.8 -0.6 -0.4 -0.2 0 0.2 0.4 0.6 0.8 1
Module 3 (MC)
rDiscoidinII
rLSLN
SSA
rDiscoidinI
rGC2
TJAI
SNA
rC14
rGRFT
rMOA
rPSL1a
TJAII
rABA
rPPL
rOrysata
rBanana
WFA
rSRL
HPA
rAAL
rGal3C
rRSL
rBC2LA
CSA
GSLII
rMalectin
rPhoSL
rPAIL
rF17AG
rCNL
rPALa
rCGL2
UEAI
rRSIIL
rCalsepa
ConA
rAOL
rPVL
rBC2LCN
-0.4 -0.3 -0.2 -0.1 0 0.1 0.2 0.3 0.4 0.5

D

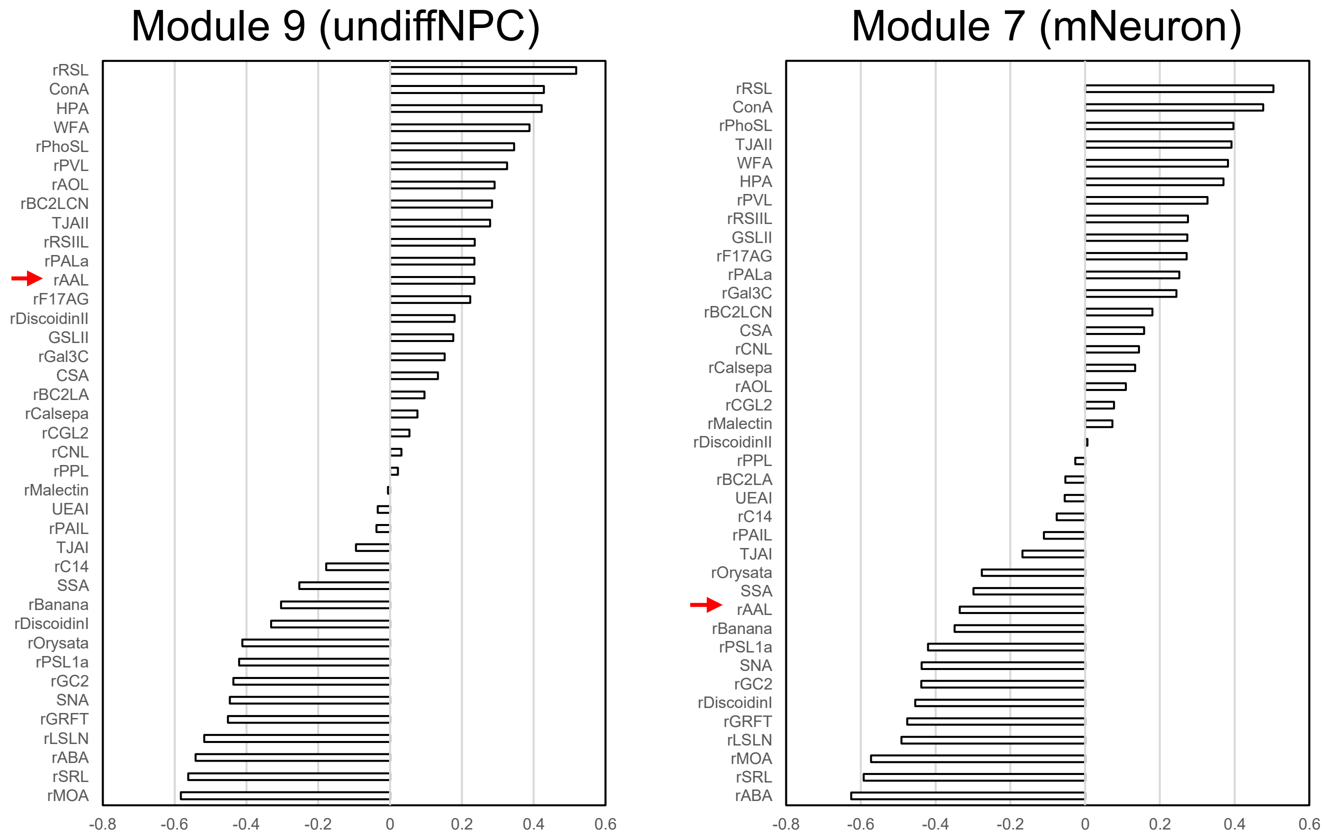

Module 9 (undiffNPC)
rRSL
ConA
HPA
WFA
rPhoSL
rPVL
rAOL
rBC2LCN
TJAII
rRSIIL
rPALa
rAAL
rF17AG
rDiscoidinII
GSLII
rGal3C
CSA
rBC2LA
rCalsepa
rCGL2
rCNL
rPPL
rMalectin
UEAI
rPAIL
TJAI
rC14
SSA
rBanana
rDiscoidinI
rOrysata
rPSL1a
rGC2
SNA
rGRFT
rLSLN
rABA
rSRL
rMOA
-0.8 -0.6 -0.4 -0.2 0 0.2 0.4 0.6
Module 7 (mNeuron)
rRSL
ConA
rPhoSL
TJAII
WFA
HPA
rPVL
rRSIIL
GSLII
rF17AG
rPALa
rGal3C
rBC2LCN
CSA
rCNL
rCalsepa
rAOL
rCGL2
rMalectin
rDiscoidinII
rPPL
rBC2LA
UEAI
rC14
rPAIL
TJAI
rOrysata
SSA
rAAL
rBanana
rPSL1a
SNA
rGC2
rDiscoidinI
rGRFT
rLSLN
rMOA
rSRL
rABA
-0.8 -0.6 -0.4 -0.2 0 0.2 0.4 0.6

E

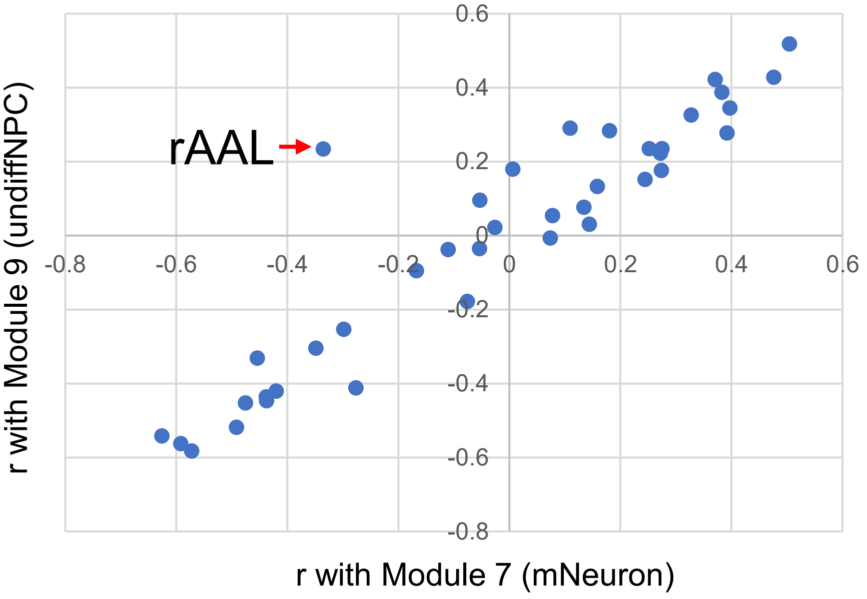

Fig. S4. Co-regulated gene module analysis.
(A) Co-regulated gene module expression plotted on UMAP coordinates for enriched subpopulations. (B) Expression data of long non-coding RNAs within Gene Module 9 were shown from the human lncRNA expression database (LncExpDB). (C) Correlation of mNPC and/or MC-enriched gene modules and lectins. The red arrow indicates rLSLN, which is highly expressed in mNPC and MC. (D) Correlation of undiffNPC or mNeuron-enriched gene modules and lectins. The red arrow indicates rAAL, which showed high binding to undiffNPC. (E) A scatterplot of Pearson correlation coefficients for each lectin with Module7 or Module9. Modules 7 and 9 show a similar lectin correlation pattern. However, rAAL stands out with a distinct correlation compared to the other lectins.

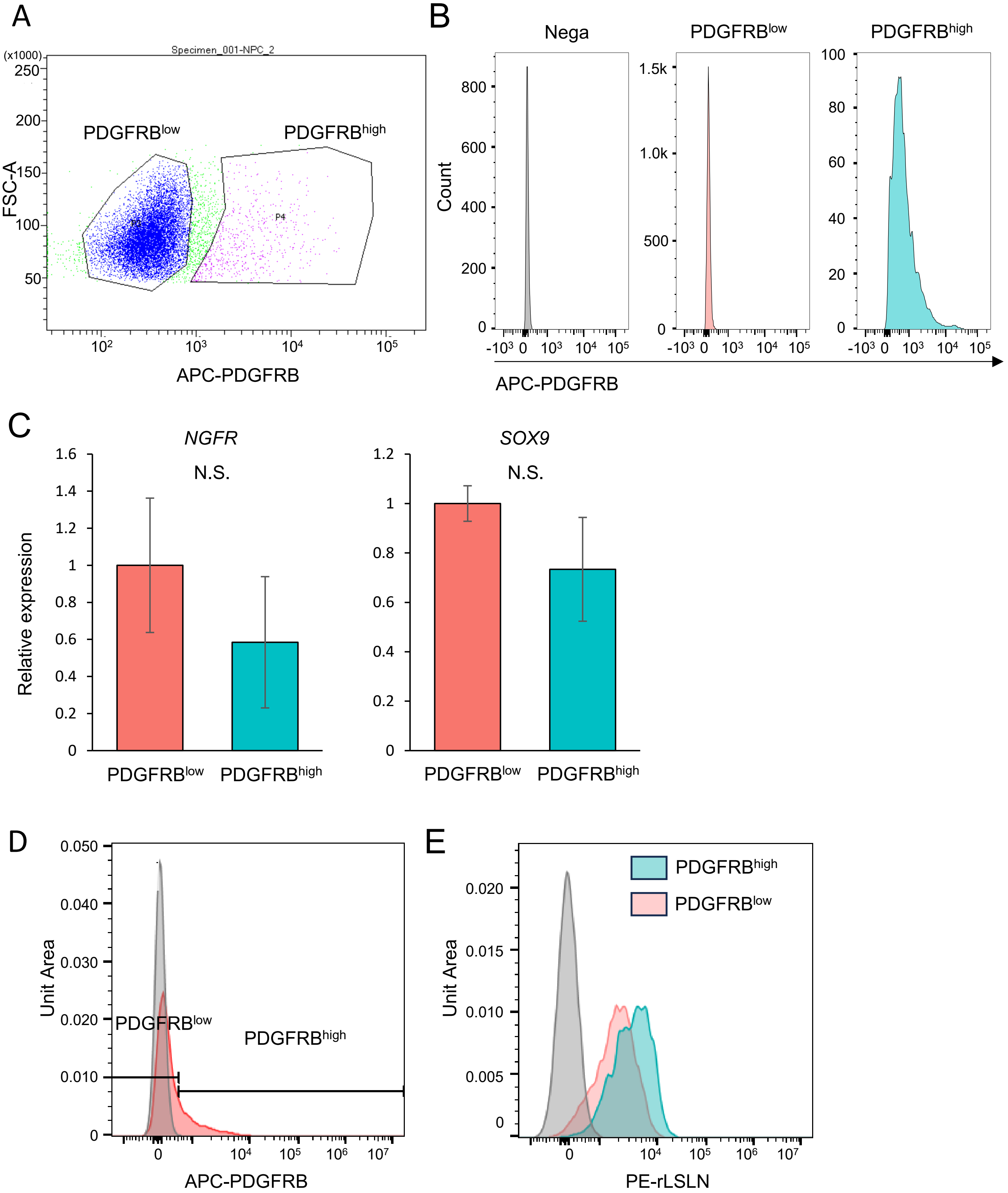

A
(x1000)
Specimen_001-NPC_2
PDGFRB^low
PDGFRB^high
P4
FSC-A
APC-PDGFRB
B
Nega
PDGFRB^low
PDGFRB^high
Count
APC-PDGFRB
C
NGFR
SOX9
N.S.
N.S.
Relative expression
PDGFRB^low
PDGFRB^high
PDGFRB^low
PDGFRB^high
D
Unit Area
PDGFRB^low
PDGFRB^high
APC-PDGFRB
E
PDGFRB^high
PDGFRB^low
Unit Area
PE-rLSLN

Fig. S5. Characterization of PDGFRB^{high} subpopulations.
(A) Fluorescence intensity of APC-conjugated PDGFRB antibody in NPCs and gating of PDGFRB^{low} and PDGFRB^{high} subpopulations for sorting. (B) Fluorescence intensity of APC-conjugated PDGFRB antibody in NPC subpopulations after sorting. (C) Quantitative PCR analysis of NCC marker gene expression (NGFR, SOX9) in PDGFRB^{low} and PDGFRB^{high} NPC subpopulations (n=3, each from an independent sorting experiment). mRNA expression levels were normalized to GAPDH. N.S., not significant. (D) Flow cytometric analysis of APC-conjugated anti-PDGFRB antibody in neural cultures. PDGFRB^{low} and PDGFRB^{high} subpopulations were defined based on fluorescence intensity. (E) Fluorescence intensity of PE-conjugated rLSLN in PDGFRB^{low} and PDGFRB^{high} subpopulations in neural cultures. The gray histogram indicates the unstained (negative control) sample.

Supplemental Methods

Neuralization of iPSCs

201B7 hiPSCs were obtained from RIKEN Bio Resource Center and maintained in mTeSR Plus medium (VERITAS, ST-100-0276) on Matrigel (Corning, 356234) coated plates. Negative mycoplasma and normal chromosomes (46, XX[20]) have been confirmed by suppliers at passage 30. For passage, Subconfluent iPSC colonies were detached by EDTA/PBS (Thermo Fisher, 11665100), dispersed by pipetting and seeded onto Matrigel-coated plates at a ratio of 1:5-1:7. iPSCs were cryopreserved by FreSR™-S (VERITAS) according to the manufacturer's protocol. All experiments were performed with passage 40-60 hiPSCs. Differentiation into NPCs was performed using STEMdiff SMADi Neural Induction Kit according to the manufacturer's protocol (VERITAS). NPCs were expanded until P4 in STEMdiff SMADi medium.
For differentiation into neurons, the NPCs were detached and dissociated into a single cell with Accutase (Innovative Cell Technologie) and seeded on polyethyleneimine (0.07%, Sigma-Aldrich) and laminin (3.3 μg/ml, FUJIFILM Wako)-coated plates. Cells were cultured with neurobasal medium (Thermo Fisher) supplemented with 2% B27 supplement (Thermo Fisher), 1% GlutaMax (Thermo Fisher), and 5 μM DAPT (Sigma-Aldrich) for 1 week. At day 7, the medium was changed to neurobasal plus medium (Thermo Fisher) supplemented with 2% B27 plus supplement (Thermo Fisher) and 1% GlutaMax. Half of the medium was changed twice a week until days 21-28.

Processing of scGR-seq data

Preprocessing of sc-RNAseq data was performed by Subio Platform (version 1.24.5849, Subio inc.) which uses fastp (version 0.22.0) for quality control of FASTQ and HISAT2 (version 2.2.0) for mapping to the human reference genome (GRCh38), and StringTie (version 2.1.1) for calculation of the raw count data. Calculation of raw count data from the FASTQ file of glycan-seq was performed by our previously developed software, Barcode DNA counting system (https://github.com/bioinfo-tsukuba/barcode-dna-counting-system) (Odaka et al., 2022).
The following data processing was performed using R (version 4.0.5). Raw count data of glycan-seq were processed with the TCC R package for normalization of glycan-seq data (Sun, Nishiyama, Shimizu, & Kadota, 2013). Both raw count and TCC-normalized data were imported into Seurat R package (version 4.0.2). To remove the low-quality cells, we set the following quality criteria, number of detected genes > 5000, number of detected genes < 30000, total number of gene count < 30000000, percentage of mitochondria DNA < 7, percentage of ribosomal genes < 7.5, and total number of lectin count < 12500. RNA data was processed using the "NormalizeData" function with the default parameters, the "FindVariableFeatures" function with the parameter selection.method = "vst", nfeatures = 2000", and "ScaleData" function with the default parameters. TCC-normalized glycan data was processed using the "FindVariableFeatures" function with the parameter "selection.method = "vst", nfeatures = 39" , and "ScaleData" function with the parameter "do.scale = FALSE, do.center = TRUE". Principal component analysis (PCA) was then performed on the RNA data and the glycan data using the "RunPCA" function with the default parameters. UMAP plot for RNA data or glycan data was calculated using the "RunUMAP" function with the parameter "dims = 1:20". For the integration of RNA and glycan data, WNN analysis was performed using "FindMultiModalNeighbors" function with the parameter "dims.list =list(1:20, 1:20), k.nn = 10, knn.range = 50", followed by "RunUMAP" function with the default parameter. Differentially expressed genes or lectins were calculated with "FindMarkers" function with the parameter "only.pos = TRUE, logfc.threshold=1" or "logfc.threshold=0", respectively. Adjusted p value for multiple comparisons was calculated using "p.adjust" function with the parameter "BH". GO enrichment analysis was performed using the DAVID 2021 (https://david.ncifcrf.gov/tools.jsp) (Huang da, Sherman, & Lempicki, 2009; Sherman et al., 2022).
For sub-clustering analysis, data from neuron or NPC samples was extracted and data processed until UMAP plot calculation was performed as described above. Clustering analysis of neurons and NPCs was then performed using the "FindClusters" function with the parameter "algorithm = 3, resolution = 1.5, verbose = FALSE" and "algorithm = 3, resolution = 0.5, verbose = FALSE", respectively. Differentially expressed genes or lectins were calculated again with the "FindMarkers" function with the parameter "only.pos =

TRUE" or "logfc.threshold=0", respectively.
For pseudotime analysis, the data from NPCs and neurons were combined and recalculated UMAP plot using "FindMultiModalNeighbors" function with the parameter "dims.list = list(1:3, 1:14), k.nn = 10, knn.range = 10", followed by "RunUMAP" function with the default parameter. The resulting UMAP plot was transfer to the "Monocle3" R package (version 1.0.1) using "as.cell_data_set" function of "SeuratWrappers" R package (version 0.3.0). Pseudotime analysis was performed using "cluster_cells" function with the default parameter, the "learn_graph" function with the parameter "use_partition = TRUE", "order_cells" function with the default parameter. Gene module was determined using the "find_gene_modules" function with the parameter "resolution=3e-2".

Quantitative analysis of fluorescence signal co-localization
To quantify the co-localization of fluorescent signals, we performed co-localization analysis using the Coloc2 plugin in Fiji (ImageJ, version 1.54p) following standard protocols. First, raw fluorescence images were converted to 8-bit grayscale and background noise was removed using the "Subtract Background" function with a rolling ball radius of 200 pixels. Subsequently, co-localization was assessed using Coloc2 with default settings. Pearson's correlation coefficient (r) and Costes' significance test (P-value) were used as primary quantitative indicators of co-localization.

Reference
Odaka, H., Ozaki, H., & Tateno, H. (2022). scGR-seq: Integrated analysis of glycan and RNA in single cells. STAR Protoc, 3(1), 101179. doi:10.1016/j.xpro.2022.101179
Huang da, W., Sherman, B. T., & Lempicki, R. A. (2009). Systematic and integrative analysis of large gene lists using DAVID bioinformatics resources. Nat Protoc, 4(1), 44-57. doi:10.1038/nprot.2008.211
Sherman, B. T., Hao, M., Qiu, J., Jiao, X., Baseler, M. W., Lane, H. C., . . . Chang, W. (2022). DAVID: a web server for functional enrichment analysis and functional annotation of gene lists (2021 update). Nucleic Acids Res, 50(W1), W216-221. doi:10.1093/nar/gkac194
Sun, J., Nishiyama, T., Shimizu, K., & Kadota, K. (2013). TCC: an R package for comparing tag count data with robust normalization strategies. BMC Bioinformatics, 14, 219. doi:10.1186/1471-2105-14-219
