# Supplementary material for: Single-cell glycome and transcriptome profiling uncovers the glycan signature of each cell subpopulation of human iPSC-derived neurons
Source: Stem Cell Reports. 2025 Sep 4;20(10):102631. doi: 10.1016/j.stemcr.2025.102631 (PMC12790714; doi:10.1016/j.stemcr.2025.102631)
Supplement: Document S2. Article plus supplemental information [file mmc14.pdf]

Stem Cell Reports
Resource
ISSCR
OPEN ACCESS

Single-cell glycome and transcriptome profiling uncovers the glycan signature of each cell subpopulation of human iPSC-derived neurons

Haruki Odaka[1] and Hiroaki Tateno[1,2,*]
[1]Cellular and Molecular Biotechnology Research Institute, National Institute of Advanced Industrial Science and Technology (AIST), Tsukuba Central 6, 1-1-1 Higashi, Tsukuba, Ibaraki 305-8566, Japan
[2]Lead contact
*Correspondence: h-tateno@aist.go.jp
https://doi.org/10.1016/j.stemcr.2025.102631

SUMMARY

Human induced pluripotent stem cell (iPSC)-derived neurons are often heterogeneous, posing challenges for disease modeling and cell therapy. We previously developed single-cell glycan and RNA sequencing (scGR-seq) to analyze the glycome and transcriptome simultaneously. Here, we applied scGR-seq to examine heterogeneous populations of human iPSC-derived neurons. We identified four subpopulations: mature neurons, immature neurons, undifferentiated neural progenitor cells (undiffNPCs), and mesenchymal cells (MCs). Lectin-binding patterns indicated high α1,3-fucose expression in undiffNPCs. MCs exhibited strong binding of a poly-LacNAc-recognizing lectin (rLSLN) and high expression of *B3GNT2*, a poly-LacNAc synthetic enzyme. Pseudotime analysis revealed that a subpopulation of NPCs acquired mesenchymal features and differentiated into MCs. Immunocytochemistry confirmed the specific detection of undiffNPCs and MCs using anti-Lewis X (α1,3-fucosylated glycan) antibodies and rLSLN. Beyond identifying cell heterogeneity, scGR-seq enables the discovery of glycan markers and detection probes for iPSC-derived cells, aiding in their further cell processing and manipulation.

INTRODUCTION

Induced pluripotent stem cells (iPSCs) are useful cell sources for disease modeling, drug screening, and regenerative medicine (Aboul-Soud et al., 2021) since they can be differentiated into any somatic cell. However, the regulation of the differentiation process of iPSCs is complex: various degrees of differentiation states and lineages of iPSCs are generated during the differentiation process. Contamination of undifferentiated cells or differentiated cells skewing from the desired lineage may confound data interpretation at disease modeling, undermine the reliability of drug screening systems, and increase the risk of tumorigenicity in cell therapy (Lee et al., 2013). Therefore, it is essential to understand the characteristics of undifferentiated and non-target cells that contaminate the target cell population and to ensure quality control by detecting and removing these cells using appropriate markers.

The surface of all living cells is decorated with glycans produced through the combined activity of hundreds of enzymes. Cell surface glycans vary depending on cell types and cellular states such as differentiation, tumorigenesis, metastatic transformation, aging, and inflammation (Fujitani et al., 2013). In particular, stem cells and tumor cells have been reported to express specific glycan epitopes that are minimally expressed in normal cells, making them promising candidate molecules for cell surface markers (Alghazali et al., 2024; Čaval et al., 2023). In fact, many stem cell surface markers such as SSEA3/4, Tra-1-60/81, and H type3 are glycans.

However, conventional glycome analytics such as mass spectrometry, high-performance liquid chromatography, and lectin microarrays require at least thousands of cells for the analysis and could not analyze the glycan expression of each cell. Therefore, it was not possible to understand the heterogeneity of complex cell populations. Recently, we have developed single-cell glycan sequencing using DNA-barcoded lectins and next-generation sequencing (scGlycan-seq) and then combined it with single-cell RNA-seq (scRNA-seq) to realize the integrated analysis of glycan and RNA in single cells (scGR-seq) (Keisham et al., 2024; Minoshima et al., 2021). The single-cell transcriptomic data allow us to analyze the gene expression of glycosylation enzymes in single cells; however, predicting cellular glycome only from gene expression data is difficult due to the complex glycosylation mechanism (Keisham et al., 2024). Together with transcriptome data, scGR-seq can also directly analyze the glycan profile of each cell from the binding signal of a panel of 39 DNA-barcoded lectins, which cover various glycan epitopes including sialylation, fucosylation, mannosylation, and galactosylation. Therefore, scGR-seq can reveal each cell's cell surface glycan expression, constituting a complex cell population, such as a tissue, and search for specific detection probes for target cells.

Human iPSC-derived neurons are expected to be sources of cell-based therapies for brain diseases such as Parkinson's disease, spinal cord injury, and stroke (Nagoshi et al., 2020; Palma-Tortosa et al., 2021; Takahashi, 2020). Despite these advantages, *in vivo* transplantation

Published by Elsevier Inc. on behalf of International Society for Stem Cell Research.
This is an open access article under the CC BY license (http://creativecommons.org/licenses/by/4.0/).

of neural progenitor cells (NPCs) from certain iPSC lines can lead to graft overgrowth and tumor formation due to the appearance of differentiation-resistant NPCs (de Luzy et al., 2021; Nori et al., 2015). Additionally, neural crest-like cell (NCC) contamination has been reported during differentiation into NPCs that generate undesired grafts after transplantation (Colleoni et al., 2010; Curchoe et al., 2010; Isoda et al., 2023). However, the glycan diversity in these undifferentiated or contaminated cells remains unknown.

In this study, we performed single-cell glycomic profiling of human iPSC-derived neurons by scGR-seq. Simultaneous analysis of transcriptome and glycan profiles has enhanced the precision of cell clustering and enabled accurate correlation of each cell type with its corresponding cell surface glycans. Two non-neural populations, undifferentiated NPCs and mesenchymal cells, were identified in iPSC-derived neurons. scGR-seq analysis has characterized the glycan profiles of these non-neuronal cells and successfully identified novel cell surface glycan markers. In conclusion, scGR-seq not only reveals the cell surface glycans of each cell comprising a complex cell population, such as iPSC-derived cells, but also allows for the discovery of detection probes that target cell surface glycans.

RESULTS

Generation of iPSC-derived neural cells

First, we differentiated iPSCs using a dual Smad inhibition strategy to generate NPCs (Figure 1A). Immunocytochemistry (ICC) showed that almost all cells were positive with pan-NPC markers, NESTIN, and PAX6 (Figure S1A). Increased expression of NPC marker genes (*NESTIN*, *PAX6*, and *SOX1*) and decreased expression of a pluripotency marker gene, *OCT4*, during neural differentiation were also confirmed by quantitative polymerase chain reaction (qPCR) (Figure S1D). NPCs were further differentiated into neurons for 21–28 days. qPCR analysis showed a robust increase of pan-neuronal (*TUBB3* and *MAP2*), telencephalon (*FOXG1*), excitatory cortical deep layer neuron (*TBR1* and *CTIP2*), and inhibitory GABAergic neuron (*GAD67*) markers by differentiation into neurons. We also detected a slight increase in cortical upper layer neuron (*CUX1*), dopaminergic neuron (*TH*), and peripheral neuron (*PRPH*) markers, but not peripheral motor neuron (*HB9*) and astroglial (*GFAP*) markers (Figures S1E and S1F). ICC confirmed the high purity of *TUJ1* (*TUBB3*-encoding neural protein)-positive neurons, and subsets of these neurons expressed *FOXG1*, *TBR1*, *CTIP2*, and *GAD67* (Figures S1B and S1C). These results demonstrated the successful differentiation of iPSC-derived NPCs and neurons, including cortical excitatory and inhibitory neurons.

scGR-seq of iPSC-derived neural cells revealed both the glycan and transcriptome signatures

To characterize the glycan expression of each cell type derived from iPSCs, we analyzed iPSCs (96 cells), NPCs (96 cells), and neurons (72 cells) by scGR-seq analysis (Odaka et al., 2022). After quality control of the single-cell data as indicated in the methods section, we obtained the scGR-seq datasets of iPSCs (81 cells), NPCs (76 cells), and neurons (49 cells), which satisfy quality criteria for downstream analysis. A median of 9,058,151 mapped reads per cell and a median of 16,273 genes per cell were detected in scRNA-seq data (Figures S1G and S1H). In scGlycan-seq, a median of 1,960 reads per cell and 30 lectins per cell were detected (Figures S1I and S1J). These RNA and glycan expression data were integrated by weighted-nearest neighbor (WNN) analysis with Seurat v4, and cells were plotted on Uniform Manifold Approximation and Projection (UMAP) based on RNA expression data, glycan expression data, or both modalities (Figure 1B) (Hao et al., 2021). In either RNA-data- or glycan-data-based UMAP plots, a small portion of cells was inadequately clustered, whereas the UMAP plot using the integrated data of two omics layers showed a clear separation of the three cell types (Figure 1B).

Next, we identified differentially expressed genes (DEGs) in each cell cluster. The highly expressed genes were as follows: iPSCs (184 genes), NPCs (97 genes), and neurons (183 genes) (Figure S1K; Table S1). These DEGs include conventional marker genes of each cell type, such as *POU5F1*, *NANOG*, and *GDF3* for iPSCs; *NES*, *PAX6*, and *SOX1* for NPCs; and *DCX*, *STMN2*, and *GAP43* for neurons (Figure S1L). To further characterize DEGs in each cell cluster, Gene Ontology (GO) enrichment analysis was performed (Table S2). DEGs in iPSCs showed enrichment of GO terms such as “amino acid transporter” and “somatic stem cell population maintenance,” suggesting its high consumption of amino acid and stemness. In NPCs, GO terms “extracellular matrix organization,” “cell migration,” and “axon guidance” were enriched. Neuron-enriched GO terms include “nervous system development” and “chemical synaptic transmission,” consistent with its neural identity.

We also identified lectins with statistically significant differential binding in each cell group from scGR-seq data (Figure 1C). The highly binding lectins were as follows: iPSCs (10 lectins), NPCs (13 lectins), and neurons (14 lectins) (Table S3). Consistent with our previous reports, the H-type3-binder (rBC2LCN) showed higher binding to iPSCs than NPCs and neurons, while the Man-binder (rBanana) and the Galβ1-3GalNAc-binders (rABA and rSRL) showed higher intensity to NPCs than the other two cell types (Minoshima et al., 2021). The core-fucose-binder (rPhoSL) showed higher intensity in

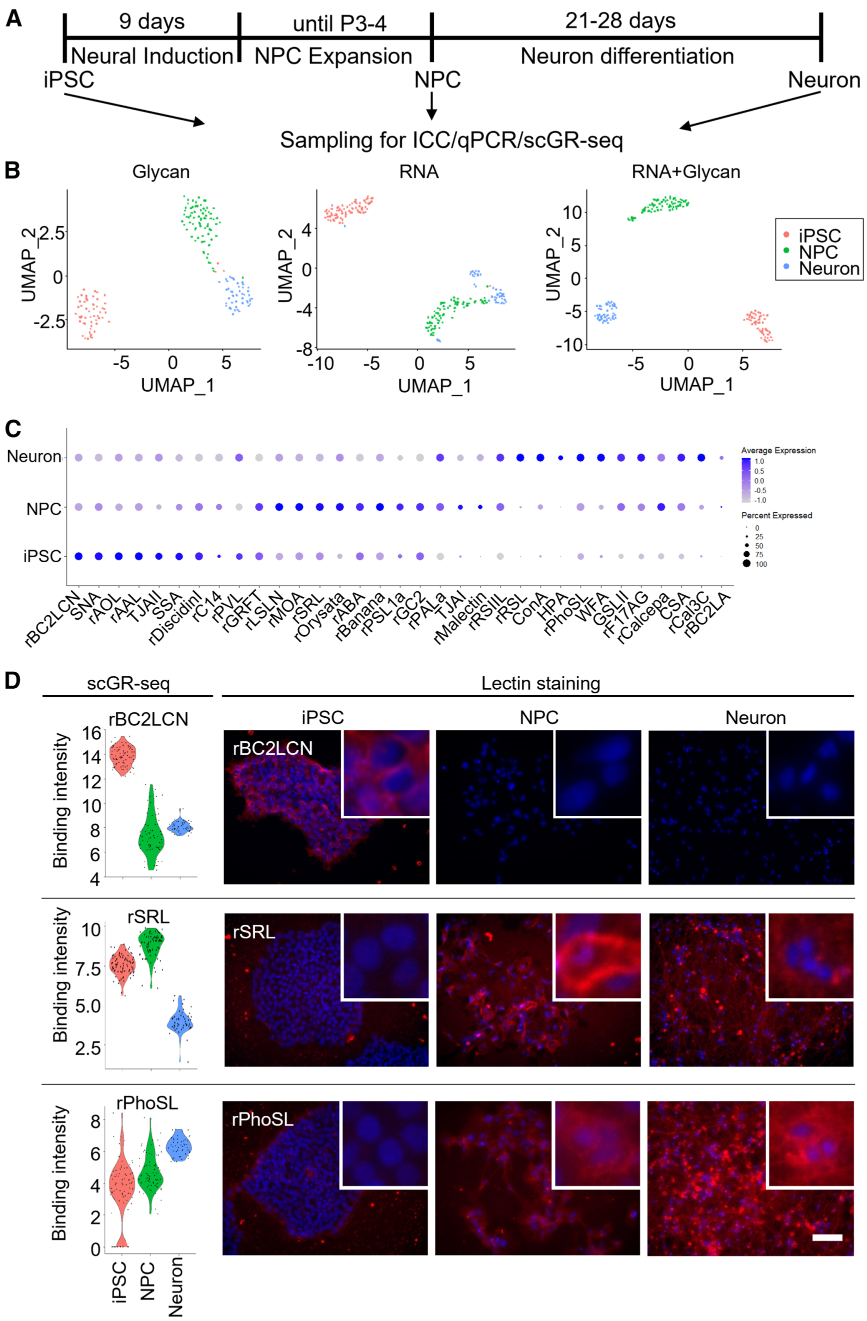

Figure 1. scGR-seq of iPSC-derived neural cells

(A) Schematic illustration of neural differentiation of iPSCs.

(B) UMAP plots using only RNA data, and glycan data, and both RNA and glycan data of iPSCs (n = 81 cells, *red*), NPCs (n = 76 cells, *green*), and neurons (n = 49 cells, *blue*).

(C) Dot plot of differentially binding lectins in each cell group.

(D) Violin plots of iPSC-binding lectin (rBC2LCN), NPC-binding lectin (rSRL), and neuron-binding lectin (rPhoSL) using scGR-seq data and fluorescent staining image with the same lectins. Insets show high magnification of selected fields. Scale bars, 100 μm.

neurons than other cells, and core-fucosylated glycans have been previously reported as brain-enriched glycan structures (Williams et al., 2022). Fluorescence staining corroborated the scGR-seq data, with high binding of rBC2LCN in iPSCs, rSRL in NPCs, and rPhoSL in neurons being confirmed by lectin staining (Figure 1D). These results showed that scGR-seq data properly illustrate the characteristic glycan and RNA expression profiles of iPSC-derived NPCs and neural cells.

Sub-clustering analysis of iPSC-derived neurons identified a unique glycan epitope in non-neural contaminants

To better understand the heterogeneity in iPSC-derived neurons, we focused on scGR-seq data originating from neuron culture samples (Figure 2A). Clustering data identified two neuron clusters, such as mature neurons (mNeuron, 15 cells) and immature neurons (imNeuron, 14 cells), and two non-neuron clusters, such as

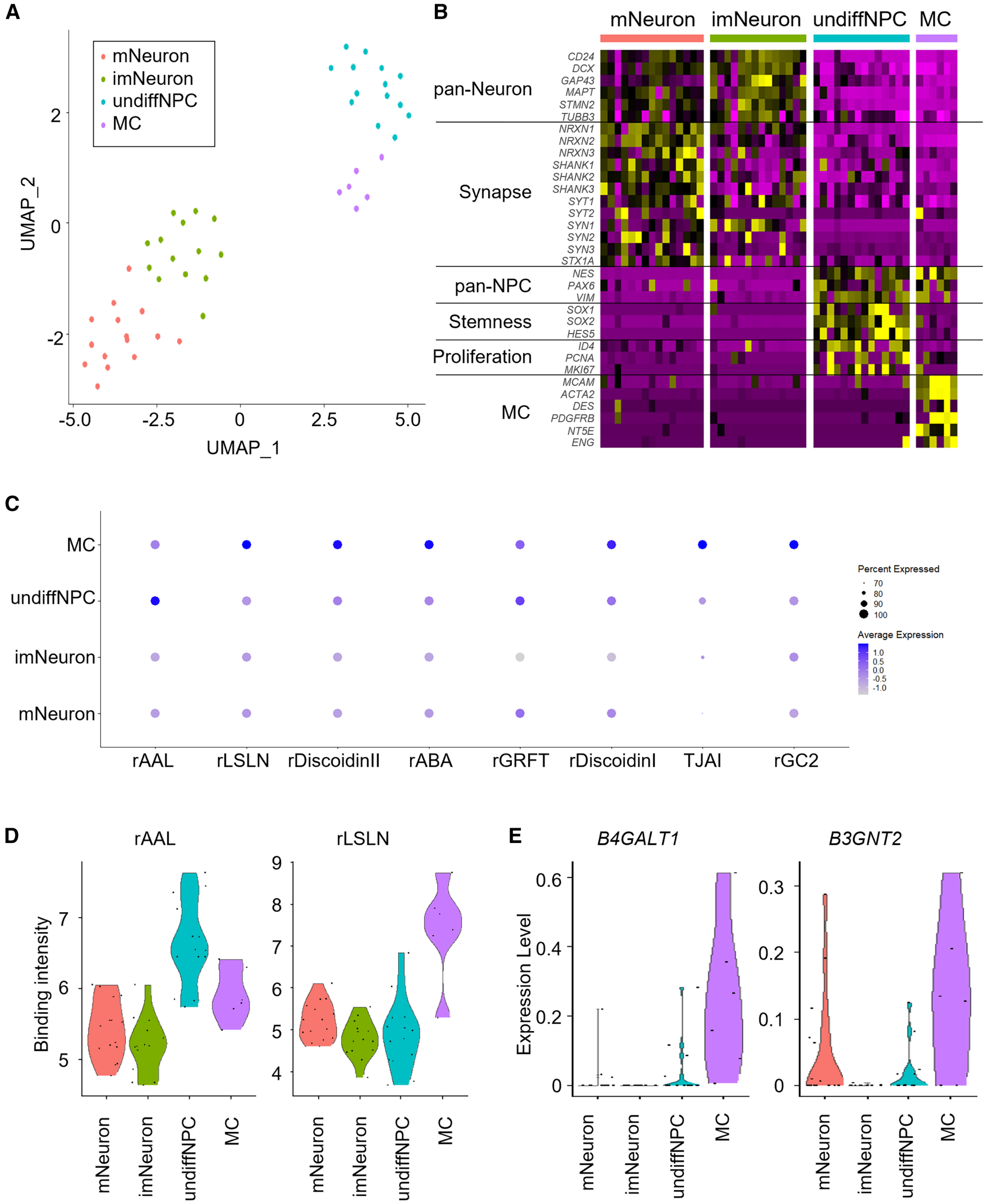

Figure 2. Sub-population analysis in iPSC-derived neurons

(A) UMAP plot using RNA and glycan data of neurons ($n = 49$). Four sub-populations are identified by clustering analysis. mNeuron ($n = 15$): mature neuron, *red*; imNeuron ($n = 14$): immature neuron, *green*; undiffNPC ($n = 14$): undifferentiated NPC, *blue*; MC ($n = 9$): mesenchymal-like cell, *violet*.

(B) Heatmap of canonical marker gene expressions in each cell cluster.

(C) Dot plot of differentially binding lectins in each cell cluster.

(D) Violin plots of undiffNPC-binding lectin (rAAL) and MC-binding lectin (rLSLN) in scGlycan-seq data.

(E) Violin plots of the mRNA expression of *B4GALT1* and *B3GNT2*.

undifferentiated NPCs (undiffNPC, 14 cells) and mesenchymal-like cells (MC, 6 cells), which were named based on their DEGs. Both mNeuron and imNeuron clusters expressed pan-neural gene markers. The expression of synaptic genes was higher in mNeuron than in imNeuron, suggesting synaptic maturation (Figure 2B; Table S4). GO enrichment analysis also showed significant enrichment of synapse-related GO terms such as "chemical synaptic transmission" in mNeurons (Table S5). On the other hand, undiffNPCs expressed pan-NPC, stemness, and proliferation markers (Figure 2B). MCs expressed pan-NPC and mesenchymal cell markers (Figure 2B). Extracellular matrix (ECM)-related GO terms, including "extracellular matrix organization" and "collagen catabolic process," were enriched in MC, suggesting a higher production of ECM. Lectins showing statistically significant differences in binding among the four sub-populations are shown in Figure 2C and Table S6. Higher binding lectins were found in undiffNPCs and MCs but not in mNeuron and imNeuron clusters. Increased binding signals were observed in the undiffNPC population for rAAL, a lectin that recognizes α1,2-, α1,3-, and α1,6-fucosylated glycans (Figure 2D). In contrast, lectins specific to α1,2-fucose (TJAII, UEA-I, and rBC2LCN) and α1,6-fucose (rPhoSL) showed no significant differences in binding among cell types. Based on this pattern, α1,3-fucosylated glycans, such as Lewis X, were considered the most likely contributors to the increased rAAL signal in undiffNPCs. Among fucosyltransferase genes, *FUT10* exhibited higher expression in the undiffNPC cluster (Figures S2A and S2B). While FUT10 was previously reported as an α1,3-fucosyltransferase involved in Lewis X synthesis in the brain (Kumar et al., 2013), recent studies have redefined it as a protein O-fucosyltransferase (Hao et al., 2025). Thus, its contribution to the increase in α1,3-fucosylation in this context remains unclear. MCs also showed higher binding of several lectins, and the most significant differentially binding lectin was a polyLacNAc-binder (rLSLN) (Figures 2C and 2D; Table S6). Consistently, MCs showed higher expression of *B4GALT1* and *B3GNT2* genes, which are involved in the poly-LacNAc elongation, suggesting that poly-LacNAc might be upregulated in this cell type (Figure 2E). To further support this observation, scatterplots illustrating the relationship between rLSLN binding and the expression levels of *B4GALT1* and *B3GNT2* are shown in Figure S2C. These plots demonstrate relatively strong correlations, with Spearman's rank correlation coefficients of 0.58 and 0.40, respectively.

iPSC-derived NPCs are composed of two subclusters, characterized by neurogenic and mesenchymal features

To further investigate the origin of sub-populations in neuron cultures, we performed a sub-population analysis in NPC cultures (76 cells). Cluster analysis identified two cell clusters in NPC cultures, neuronal NPCs (nNPCs, 45 cells), and mesenchymal-like NPCs (mNPCs, 31 cells) (Figure 3A). Identification of the DEGs between the two clusters showed that nNPCs were characterized by higher expression of early neural genes such as *DCX*, *MAP1B*, and *HES6*, while mNPCs highly expressed mesenchymal genes such as *ACTA2*, *PDGFRB*, and *MCAM* (Figure 3B; Table S7). Note that both nNPCs and mNPCs expressed a comparable level of pan-NPC, stemness, and proliferation marker genes, suggesting that these clusters cannot be distinguished by canonical NPC markers (Figure S3). GO enrichment analysis for DEGs showed significant enrichment of "nervous system development" in nNPCs, supporting its commitment to differentiation into neurons (Table S8). On the other hand, mNPCs showed significant enrichment of ECM-related GO terms, including "extracellular matrix organization," which were also enriched in the MC cluster. rPhoSL, which showed higher binding to neurons than iPSCs and NPCs (Figure 1C), also showed higher binding to nNPCs than mNPCs, as expected (Figures 3C and 3D; Table S9). Consistently, mNPCs showed a higher signal in rLSLN and higher expressions in *B4GALT1* and *B3GNT2* genes than nNPCs, similar to MCs (Figures 3C–3E).

In previous studies, it was found that NCCs in iPSC-derived NPCs could become contaminated and potentially differentiate into mesenchymal cells (Colleoni et al., 2010; Curchoe et al., 2010; Isoda et al., 2023). We examined the expression of NCC marker genes (*SOX9*, *SOX10*, *PAX3*, *PAX7*, and *NGFR*) in mNPCs. Unexpectedly, mNPCs showed lower expression of these NCC marker genes than nNPCs (Figure S7). *PAX3* and *PAX7* were not detectable in mNPCs and nNPCs. Furthermore, we observed a strong expression of *PAX6*, a negative marker of NCC, in mNPCs. This led us to conclude that mNPCs are not NCCs, but NPCs that have acquired mesenchymal features.

Integrated analysis of RNA and glycans with pseudotime revealed the dynamic changes in glycan and transcriptome signatures along the cell trajectory

Next, we performed pseudotime analysis to clarify the relationship between neural sub-populations. The cells from NPCs and neuron cultures were plotted on a UMAP plot based on both glycan and RNA modalities and then their trajectory was determined by Monocle 3 (Figures 4A and 4B). Pseudotime analysis identified two distinct lineage trajectories: the neuron lineage trajectory and the MC lineage trajectory. The neuron lineage trajectory consists of the sequential transitions from nNPCs and undiffNPCs to imNeuon/mNeurons, illustrating differentiation from NPCs to neurons. The MC lineage trajectory, on the other hand, consisted of transitions from nNPCs and mNPCs to

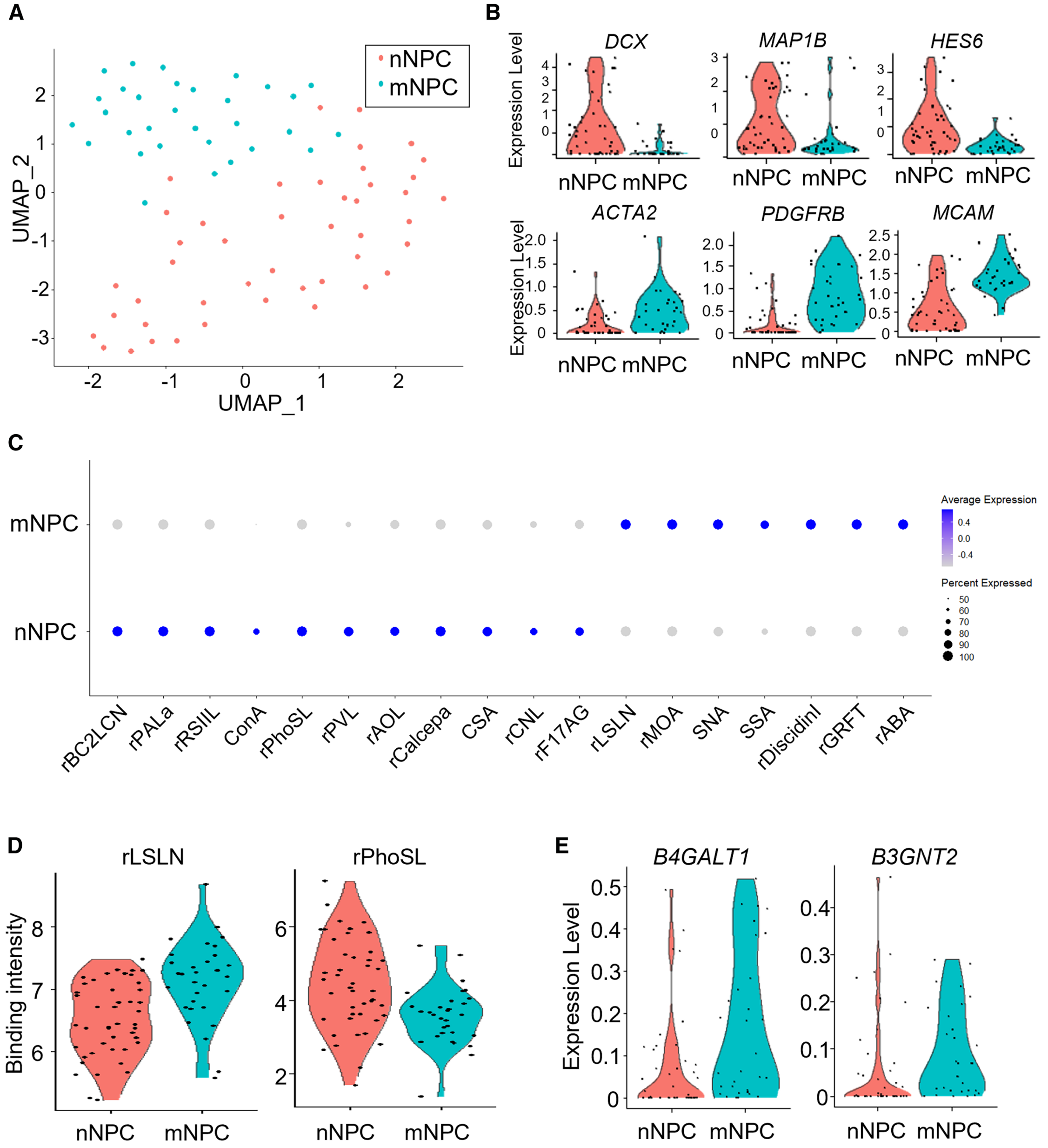

Figure 3. Sub-population analysis in iPSC-derived NPCs

(A) UMAP plot using RNA and glycan data of NPCs (n = 76). Two sub-populations are identified by clustering analysis. nNPC (n = 45): neuronal NPC, *red*; mNPC (n = 31): mesenchymal NPC, *green*.
(B) Violin plots of neuronal and mesenchymal gene expressions in each cell cluster.
(C) Dot plot of differentially binding lectins in each cell cluster.
(D) Violin plots of mNPC-binding lectin (rLSLN) and nNPC-binding lectin (rPhoSL).
(E) Violin plots of the mRNA expression of *B4GALT1* and *B3GNT*.

MCs. These results supported the idea that nNPCs and mNPCs are the progenitors of neurons and MCs, respectively.

Simultaneous measurements of glycan and RNA in single cells provide a means to investigate the relationship between gene expression profile and glycan profile. To summarize the gene expression profile in NPC and neuron culture datasets, co-regulated genes are grouped into gene modules by Monocle 3 (Cao et al., 2019). Twelve gene modules were obtained and examined expression in each cell cluster and enrichment of GO terms to characterize gene modules (Figures 4C and S4A; Table S10). Modules 1, 2, 4,

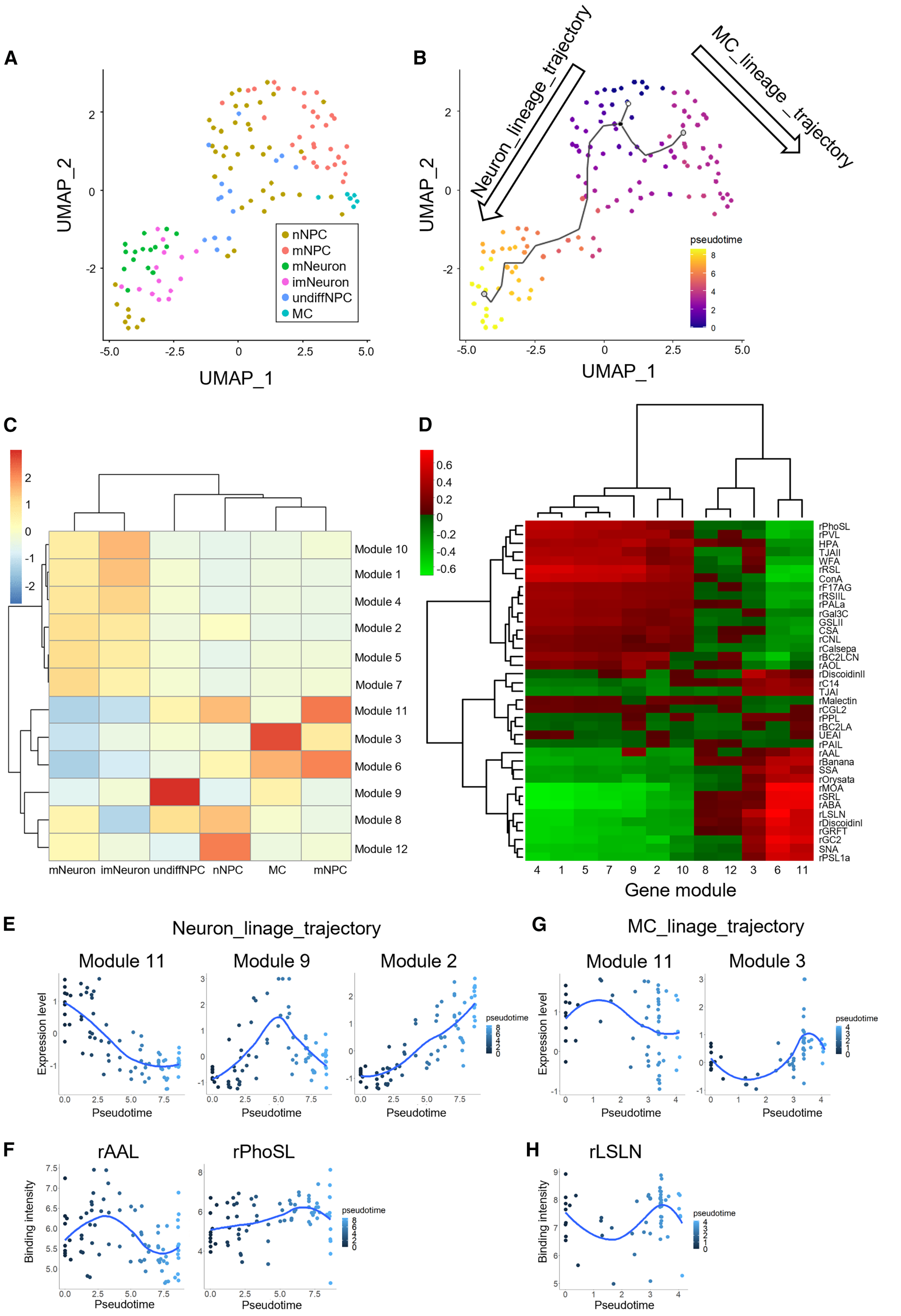

(legend on next page)

5, and 7 were highly expressed in the imNeuron/mNeuron cluster and showed enrichment of neuron-related GO terms, including “nervous system development” and “chemical synaptic transmission.” Module 10 is also expressed in a portion of the imNeuron/mNeuron cluster and shows GO term enrichment “response to bacterium,” consisting of *C3* and *DHX58* genes (Figure S4A; Table S10). Module 11 comprises cell cycling genes annotated as “cell division” and is highly expressed in proliferating cells such as mNPCs/nNPCs. Modules 3 and 6 showed enrichment of ECM-related GO terms such as “cell adhesion” and were enriched in MC and mNPC clusters, respectively. Module 9 is highly specific in the undiffNPC cluster and includes several cancer-associated long non-coding (lnc) RNA genes (*CASC15*, *NFIA-AS2*, *LINC01748*, *LINC00689*, *DNAJC27-AS1*, and *FEZF1-AS1*) that are expressed in neuroblastoma, glioma, and other cancers (Liu et al., 2019; Russell et al., 2015; Xin et al., 2020; Zhi et al., 2015). The expression database of human lncRNAs (LncExpDB) showed that these lncRNA genes are also highly expressed in the embryonic brain but merely detected in the postnatal brain (Figure S4B) (Li et al., 2021). Modules 8 and 12 were highly expressed in nNPC and showed enrichment of neurogenesis-related and signaling-cascade-related terms.

We next examined the correlation between gene modules and the binding profile of lectins (Figure 4D). Based on the correlation pattern with lectins, gene modules were mainly separated into two clusters, one consisting of modules 1, 2, 4, 5, 7, 9, and 10, and another consisting of modules 3, 6, 8, 11, and 12. Modules 1, 2, 4, 5, 7, 9, and 10 were enriched in mNeuron, imNeuron, and undiffNPC, while modules 3, 6, 8, 11, and 12 were enriched in nNPCs, mNPCs, and MCs, suggesting the similarity of glycan profile within each cluster. Both modules 3 and 6, which were enriched in mNPCs and MCs, showed a high correlation with the poly-LacNAc-binder rLSLN, consistent with its high binding with mesenchymal populations (Figure S4C). Although neuron/imNeuon-enriched module 7 and undiffNPC-enriched module 9 showed similar lectin correlation patterns, the Fuc-binder rAAL stands out with a distinct correlation compared to the other lectins, supporting the potential usefulness of glycan ligands of rAAL as a marker for undiffNPC (Figures S4D and S4E).

Pseudotime analysis with scGR-seq also highlighted unique genes and lectins that showed differential expression along the cell trajectory. In the neuron lineage trajectory, gene module 11 was gradually decreased while gene module 2 was gradually increased (Figure 4E). Gene module 9 was spiked in the middle, then decreased after neuralization (Figure 4E). In addition, the core-Fuc binder rPhoSL was increased in the late stage, while the Fuc-binder rAAL showed a high peak at the middle stage, followed by a sharp decrease in the late stage (Figure 4F). In the MC lineage trajectory, the gene module 11 was high in the middle stage (mNPC) while the gene module 3 was increased in the late stage (MC) (Figure 4G). As expected, the mNPC/MC marker lectin rLSLN was also increased in the middle to late stage (Figure 4H). Taken together, the integrated analysis of glycan and RNA revealed its lineage identity and improved understanding of correlations and expression dynamics of the two modalities.

Identification of sub-populations in iPSC-derived NPCs and neurons through the expression of cell surface markers

Finally, we examined whether identified markers can detect sub-populations found in scGR-seq. Due to the relatively low fold change of differentially binding lectin between nNPCs and mNPCs (Figure 3), we searched the membrane-protein-encoding genes that can distinguish two populations in NPCs. Receiver operating characteristic (ROC) curve analysis identified that the *PDGFRB* gene showed a high performance in distinguishing mNPCs and nNPCs (AUC = 0.882) (Table S7). Flow cytometry analysis with PDGFRB antibody showed that a subpopulation of iPSC-derived NPCs highly expressed PDGFRB (Figure 5A). These PDGFRB^{high} NPCs also showed higher binding with rLSLN than PDGFRB^{low} NPCs, consistent with the glycan profile of mNPCs (Figure 5A). We then

Figure 4. Pseudotime trajectory analysis with scGR-seq

(A) UMAP plot using RNA and glycan data of NPCs (n = 76) and neurons (n = 49). The graph shows the identified sub-populations in Figures 2 and 3.

(B) UMAP plot with pseudotime in each cell with a branched trajectory graph inferred by Monocle 3. The neuron lineage trajectory consists of sequential transitions from nNPCs and undiffNPCs to imNeuon/mNeurons. The MC lineage trajectory consisted of transitions from nNPCs and mNPCs to MCs.

(C) Heatmap plots of co-regulated gene modules in each cell cluster.

(D) Heatmap plot of the correlation between co-regulated gene modules and the binding profile of lectins.

(E and F) Dynamically regulated gene modules (E) and lectins (F) along the pseudotime of neuron-linage trajectory. The x axis represents the pseudotime of the neuron-linage trajectory.

(G and H) Dynamically regulated gene modules (G) and lectins (H) along the pseudotime of MC-linage trajectory. The axis represents the pseudotime of the MC-linage trajectory.

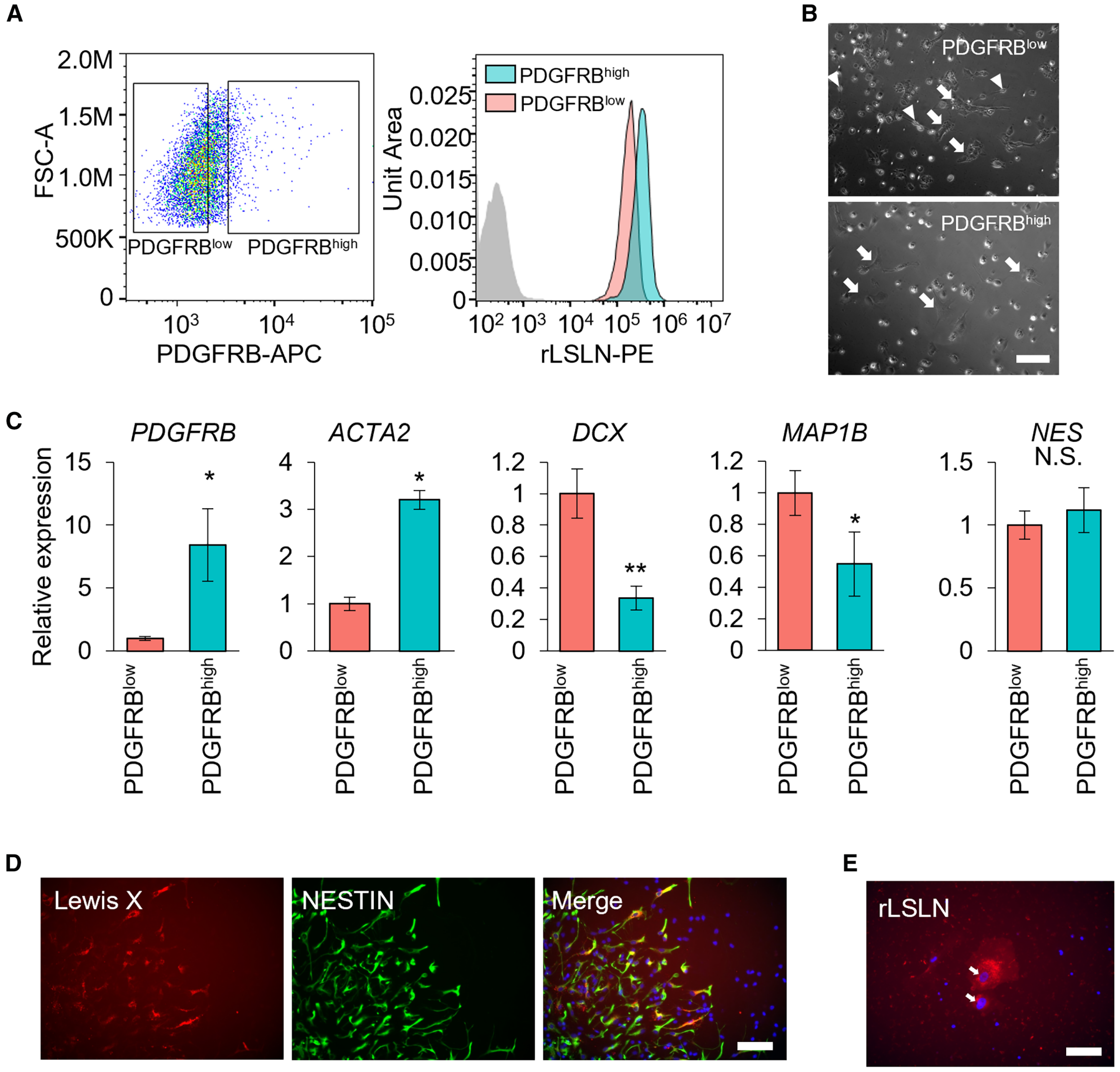

Figure 5. Fluorescence staining of sub-population in iPSC-derived NPCs and neurons

(A) Flow cytometric analysis of PDGFRB expression and binding to rLSLN in iPSC-derived NPCs. The left panel shows the fluorescence intensity of APC-conjugated PDGFRB antibody and the gating of PDGFRB^{low} and PDGFRB^{high} subpopulations. The right panel shows the fluorescence intensity of PE-conjugated rLSLN in PDGFRB^{low} and PDGFRB^{high} subpopulations.

(B) Phase-contrast image of PDGFRB^{low} and PDGFRB^{high} NPCs after isolation with FACS. Arrows in PDGFRB^{low} cells show colony-formed epithelial-like cells, while arrowheads show polarized cells. Arrows in PDGFRB^{high} cells show flat cells. Scale bars, 100 μm.

(C) qPCR analysis of mesenchymal and neural marker gene expressions in PDGFRB^{low} and PDGFRB^{high} subpopulations ($N = 3$, each from an independent sorting experiment). Relative mRNA expression of mesenchymal markers (*PDGFRB* and *ACTA2*), neural markers (*DCX* and *MAP1B*), and NPC marker (NES) were shown. Expression levels of each marker were normalized with *GAPDH* expression. Mean ± SD. *$p <$ 0.05, **$p < 0.01$. N.S.: not significant.

(D) Expression of Lewis X and NESTIN in a subpopulation of iPSC-derived neuron culture. Scale bars, 100 μm.

(E) rLSLN-positive non-neural cells in iPSC-derived neuron culture. The arrow indicates rLSLN-positive large flat cells. Scale bars, 100 μm.

isolated PDGFRB^{low} and PDGFRB^{high} NPCs with a cell sorter (Figures S5A and S5B). PDGFRB^{low} NPCs consisted of colony-formed epithelial-like cells and polarized cells, which were reminiscent of neuroepithelial cells and neuroblasts, respectively (Figure 5B). On the other hand, PDGFRB^{high} NPCs were composed of flat cells, which were reminiscent of mesenchymal cells (Figure 5B). qPCR analysis confirmed higher expression of mNPC marker genes (*PDGFRB, ACTA2*) and lower expression of nNPC marker genes (*DCX, MAP1B*) without alternation in pan-NPC marker (*NES*) in PDGFRB^{high} NPCs (Figure 5C). In addition, NCC marker genes (*NGFR* and *SOX9*) were not

enriched in PDGFRB^{high} NPCs, and *SOX10* expression was undetectable in both PDGFRB^{low} and PDGFRB^{high} NPCs, consistent with the results of scGR-seq analysis (Figure S5C). These results guarantee the existence of mNPC and nNPC sub-populations in NPC cultures.

Next, we examined whether the identified glycan markers can detect non-neural populations (undiffNPC and MC) in iPSC-derived neurons. Increased binding signals were observed in the undifferentiated NPC (undiffNPC) population for rAAL, a lectin known to recognize α1,2-, α1,3-, and α1,6-fucosylated structures, whereas no significant changes were detected for lectins specific to α1,2-fucose (TJAII, UEA-I, and rBC2LCN) or α1,6-fucose (rPhoSL). Based on this, we tested Lewis X, an α1,3-fucosylated glycan, as the most plausible candidate. ICC showed the presence of NESTIN and Lewis X double-positive undiffNPCs in iPSC-derived neuron cultures (Figure 5D). To further quantify their spatial association, we performed co-localization analysis using the Coloc 2 plugin. Eight microscopic fields consistently showed statistically significant colocalization between NESTIN and Lewis X signals, with a mean Pearson's correlation coefficient of 0.414 ± 0.119 and a Costes p value of 1.00 ± 0.00 ($N = 8$), indicating non-random co-localization (threshold for significance: Costes p value >0.95). On the other hand, MCs showed a high signal of the poly-LacNAc-binder (rLSLN) and high expression of *B4GALT1* and *B3GNT2* genes, which mediate poly-LacNAc elongation (Figure 3D). Lectin staining determined rLSLN-positive large flat cells, which are similar to mNPC morphology (Figure 5E). Furthermore, we validated rLSLN binding in MCs by flow cytometry, confirming that PDGFRB^{high} MC subpopulations exhibit elevated rLSLN signals (ΔMFI of rLSLN: PDGFRB^{low}: 1843.3, PDGFRB^{high}: 3599.3) (Figures S5D and S5E). These results demonstrated the presence of two types of non-neuronal cells, undiffNPCs and MCs, detectable by cell surface glycans, in iPSC-derived neural cell cultures.

DISCUSSION

Cell surface glycans such as SSEA3/4, Tra-1-60/81, and H type3 are widely used as pluripotent stem cell markers (Alghazali et al., 2024; Hirabayashi et al., 2015). While conventional glycan analysis provides only for bulk-level samples, the recently developed scGR-seq enables simultaneous glycan and RNA profiling at the single-cell level (Minoshima et al., 2021). This allows identification of glycan profiles in heterogeneous populations and may facilitate the development of cell surface markers for purifying therapeutic cells derived from iPSCs.

To test this, we applied scGR-seq to iPSC-derived neurons, potential sources for treating brain diseases. Integrated glycan and RNA analysis in single cells distinguished iPSCs, NPCs, and neurons into clusters consistent with sample origins. Marker genes (e.g., *POU5F1*, *NES*, and *DCX*) and lectins (e.g., rBC2LCN, rSRL, and rPhoSL) matched previously reported markers. Sub-clustering revealed heterogeneity in NPCs and neurons. We identified poly-LacNAc expression in mesenchymal cells (detected by rLSLN) and proliferating undifferentiated NPCs marked by Lewis X glycan and stained with anti-Lewis X antibody. These findings demonstrate that single-cell glycan and RNA profiling enables unbiased identification of subpopulations and their surface glycan markers.

Differentiation-resistant NPCs may cause uncontrolled NPC expansion, reducing neuronal yield and increasing tumorigenicity risk in transplantation (Nori et al., 2015). Here, we found the differentiation-resistant NPC clusters in iPSC-derived neurons, which retained robust expression of stemness and proliferation marker genes and cancer/embryo-associated lncRNA. While their glycan profiles resembled those of imNeuron/mNeuron clusters, undiffNPCs showed notably high Lewis X expression. This epitope is also known to be enriched in neural stem/progenitor cells in the embryonic mouse brain ventricular zone (Kumar et al., 2013) and in human ESC-/iPSC-derived neural stem cells (Yuan et al., 2011). These findings support Lewis X as a potential glycan marker of NPC stemness.

Glycosyltransferase genes are often expressed at low levels and thus may not be consistently captured in scRNA-seq datasets with lower sequencing depth. In this study, we took advantage of the high sequencing depth afforded by plate-based scRNA-seq—compared to droplet-based methods—which enabled more reliable detection of glycosyltransferases to allow for a direct comparison between glycosyltransferase mRNA expression and lectin-binding signals at the single-cell level. For example, poly-LacNAc—the glycan epitope recognized by rLSLN and enriched in the MC cluster—showed a strong positive correlation with the transcript levels of its biosynthetic enzymes *B4GALT1* and *B3GNT2*. This finding suggests that, at least for certain glycan structures, glycosyltransferase mRNA expression can be a major determinant of glycan abundance. In contrast, this relationship was not observed for the Lewis X epitope, which was enriched in undiffNPCs. Canonical α1,3-fucosyltransferases—enzymes typically responsible for Lewis X biosynthesis—did not show increased expression in undiffNPCs. Among the fucosyltransferase genes, *FUT10* was the only enzyme that exhibited elevated gene expression. While it had previously been proposed as a Lewis X-synthesizing enzyme in the brain (Kumar et al., 2013), recent studies have reclassified FUT10 (along with FUT11) as protein O-fucosyltransferases rather than α1,3-fucosyltransferases (H. Hao et al., 2025). This discrepancy highlights that glycosyltransferase transcript levels do not always

predict the levels of their associated glycan products. Indeed, cell-surface glycan expression is governed by a complex, multilayered regulatory network, including substrate availability, precursor supply, correct intracellular localization of enzymes, and the abundance of carrier glycoproteins at the plasma membrane. As a result, glycosyltransferase mRNA levels do not always correlate with the abundance of their associated glycan products. Therefore, while glycosyltransferase mRNA expression can contribute to glycan expression in certain cases, it is not sufficient to fully predict glycosylation profiles. In this regard, our lectin-based profiling approach offers a valuable and complementary strategy for characterizing glycosylation at the product level. By quantifying glycan epitopes directly through lectin-binding intensity, this method provides important insights into the glycome that cannot be inferred from transcriptomics alone.

A previous study reported that transplantation of iPSCs or fetal-derived NPCs into the brain or spinal cord causes histologically distinct tissues, differentiating neural tissue (DNT), undifferentiated neural tissue (UDNT), blastemal tissue (BLT), and benign mesenchymal tissue (Sugai et al., 2016). Interestingly, major component cells of these tissues seem to correspond with sub-populations in our neuron culture, such as mNeurons/imNeurons for DNT, undiffNPC for UDNT/BLT, and MC for benign mesenchymal tissue, suggesting *in vitro* differentiation mimics the differentiation after *in vivo* transplantation. Considering that immature undifferentiated tissues such as UDNT/BLT could contribute to the overgrowth of transplants and the transformation into embryonal tumors, evaluation of the emergence of undiffNPCs *in vitro* culture may reflect the risk of tumorigenicity of iPSC-derived NPCs. Although the detailed mechanism of the differentiation resistance of NPCs was still unclear, it has been reported that the overgrowth of NPC transplants is correlated with an increased number of copy-number variants and abnormal karyotypes of iPSCs (Sugai et al., 2016). Such genetic instability has occurred during the reprogramming process and long-term culture of iPSCs. In this study, iPSCs with passages 40–60 were used, which may have induced genetic instability and resulted in the appearance of undiffNPCs in neural cultures.

The appearance of mesenchymal cells has been reported in a variety of iPSC-derived neural culture systems such as 2D culture, transplanted NPCs, and cerebral organoids (Curchoe et al., 2010; Fair et al., 2020; Sugai et al., 2016). It has been believed that the mesenchymal cells originated from the contaminated NCCs (Colleoni et al., 2010; Curchoe et al., 2010; Sugai et al., 2016). Here, we found the contamination of mNPCs that retained both NPCs and mesenchymal features in iPSC-derived NPCs and neurons. Pseudotime analysis showed a continuous transition trajectory from nNPCs and mNPCs toward MCs, suggesting that mesenchymal characteristics may emerge progressively during differentiation. scGR-seq analysis revealed high expression of poly-LacNAc as a novel marker for mesenchymal subpopulations within the NPC/Neuron cultures. An increase in poly-LacNAc has been previously reported during the epithelial-mesenchymal transition (EMT) in cancer cells, suggesting that this glycan change is similarly associated with the EMT from neuroepithelial cells to mNPC/MC populations (Lucena et al., 2016). Consistent with our findings, Isoda et al. Recently reported the existence of an NCC-like NPC subpopulation in iPSC-derived NPCs, expressing both NPC markers (PSA-NCAM) and mesenchymal markers (CD73 and CD105) (Isoda et al., 2023). These NCC-like NPCs differ from our mNPCs by exclusively expressing the NCC marker SOX9 but share several key characteristics, including the expression of PDGFR and mesenchymal markers. Interestingly, it has been reported that PDGFD-PDGFRB is highly expressed in the germinal regions of the human dorsal neocortex but not in mice (Lui et al., 2014). Human-specific PDGFRB signaling contributes to the proliferation of neocortical radial glia (RGs) and may have played a role in the evolutionary expansion of the human neocortex. Although it is unclear whether these PDGFRB-positive RGs express other mesenchymal markers, mNPCs may reflect these human-specific RG subtypes. It will be necessary to investigate whether the acquisition of mesenchymal features of NPCs also occurred in CNS development or is artificial for *in vitro* differentiation of iPSC-derived NPCs.

This study has certain limitations. First, although lectin-based glycan profiling methods enable the characterization of glycan epitopes, they do not facilitate the identification of full glycan structures. Second, we utilized plate-based scGR-seq, which limits the number of analyzed cells to a few hundred, potentially leading to the omission of extremely rare cell populations due to the small sample size. However, given the inherent trade-off between sample size and sequencing depth, our analysis achieved an exceptionally high sequencing depth, with a median of 9,058,151 transcript reads per cell. Notably, we successfully detected glycosyltransferases, such as fucosyltransferases, which are typically challenging to identify in single-cell analysis due to their low expression levels. To compensate for the smaller number of cells and to enhance the robustness of our findings, we additionally validated key observations using complementary methods, including flow cytometry, fluorescence staining, and qPCR. These independent validation experiments confirmed the trends observed in the single-cell dataset, thereby strengthening the reliability of our conclusions. Recently, we developed a droplet-based scGR-seq platform capable of processing 10,000 cells simultaneously (Keisham et al., 2024). This method could be applied to investigate the rare cell

populations present in iPSC-derived neuron cultures in future studies.

scRNA-seq enables the identification of cell types and their RNA markers in a non-invasive manner and has been widely used to identify markers in various cell subpopulations. While RNA analysis of membrane-protein-encoding genes has been employed to search for cell surface markers for detection and cell sorting, these markers cannot always be reliably identified through single-cell RNA analysis due to its generally low sequencing depth. Our study demonstrated that scGR-seq can be used to analyze cellular heterogenicity and determine the cell marker genes as well as the cell surface glycans. The integration of multiple modalities, glycans and RNA, can further enhance the precision of cell clustering. By conducting integrated analyses of glycan and RNA modules, the biological functions of RNA and glycans within each cell population can be inferred. Although several single-cell glycan analysis methods have been reported, scGR-seq is the only method capable of profiling diverse glycan antigens alongside RNA information (Dworkin et al., 2022; Kearney et al., 2021; Ma et al., 2022). scGR-seq can apply to any type of cells including iPSC-derived cells, organoids, tissue, and tumors. Identified cell surface glycan markers can be targeted by lectin or antibody to detect, isolate, or remove target cells. Therefore, the scGR-seq analysis provides the means to manipulate sub-populations in heterogenic cells, which will contribute to the control of the quality and safety of iPSC-derived cell products.

MATERIALS AND METHODS

scGR-seq of iPSCs, NPCs, and neurons

scGR-seq analysis was performed as described previously (Odaka et al., 2022). Details of the differentiation protocol from iPSCs to neurons are provided in the supplemental information. Briefly, iPSCs and NPCs were detached and dissociated for single cells with Accutase. For dissociation of neurons, Accutase supplemented with papain (50 units/mL, Worthington) and L-cysteine (0.25 mg/mL, Sigma-Aldrich) was applied; 1×10^5 cells were incubated with 1% BSA-PBS containing DNA-oligonucleotide-conjugated lectin library (0.5 μg/mL) at 4°C for 1 h. The cells were washed with 1% BSA-PBS three times, and single cells were manually picked into a PCR tube with 10 μL distilled water using the TOPick I Live Cell Pick system (Yodaka Giken). The cells were irradiated at 365 nm, 15 W for 15 min using UVP Blak-Ray XX-15L UV Bench Lamp (Analytik Jena) and centrifuged at top speed for 30 s. Supernatants containing DNA barcodes were transferred to new PCR tube, and cells were lysed with lysis buffer of GenNextRamDA-seq Single Cell Kit (TOYOBO). DNA barcode samples were amplified by PCR with i5/i7 index primer and NEBNext Ultra II Q5 Master Mix (New England Biolabs) for 20 cycles. All PCR products were combined into one tube and purified by Agencourt AMPure XP (Beckman Coulter.). The quality of PCR products was confirmed by MultiNA (Shimadzu) and sequenced by the MiSeq sequencer (26 bp, paired-end) (Illumina). RNA library for scRNA-seq was prepared by GenNextRamDA-seq Single Cell Kit according to the manufacturer's protocol. The quality of the RNA library was analyzed by MultiNA and sequenced by the Nova-Seq 6000 (151 bp, paired-end, NPCs, and iPSCs) and HiSeqX (151 bp, paired-end, neurons). Details of scGR-seq data processing are provided in the supplemental information.

Fluorescence staining

The cells were fixed with 4% paraformaldehyde at room temperature for 20 min. For intracellular antigen detection, the cells were incubated with 1% BSA-PBS containing 0.2% Triton X-100, whereas for extracellular antigen detection, the cells were incubated with 1% BSA-PBS at room temperature for 30 min. The cells were then stained with primary antibody or lectin at 4°C for overnight. After washing the cells three times with PBS, secondary antibodies were added as needed. Following another three washes with PBS, Hoechst33342 (1 μg/mL, 346–07951, Fujifilm Wako) was added and incubated at room temperature for an hour. After washing once with PBS, images were taken under an inverted fluorescence microscope (IX51, Olympus). The information on the antibodies used is provided in Table S11. Details of the quantitative analysis of fluorescence signals are provided in the Supporting Information.

Quantitative RT-PCR

Total RNA was extracted from the samples using the Rneasy Mini Kit (QIAGEN), following the manufacturer's protocol. The extracted RNA was then converted into cDNA using the QuantiTect Reverse Transcription Kit (QIAGEN). qRT-PCR was performed using PowerUp SYBR Green Master Mix (Thermo Fisher) and CFX Connect (Bio-Rad). The mRNA expressions of the specified genes were normalized to the expression of the GAPDH gene. The primer sequence for qRT-PCR was described in Table S12.

Fluorescence-activated cell sorting

The NPCs were detached and dissociated into a single cell with Accutase. After washing the cells with 1% BSA/PBS, PE-labeled rLSLN (1/100) and APC-labeled anti-PDGFRB antibody (1/20, BioLegend, 323608) were added and incubated on ice for an hour. The cells were washed twice with 1% BSA/PBS and were analyzed using CytoFLEX (Beckman Coulter) for analysis or BD FACSAria III (BD biosciences) for cell sorting.

Statistics

Statistical analysis was performed using Seurat v4 for scGR-seq data and EZR on R commander version 1.41 for other analyses (Kanda, 2013). Unless otherwise specified, non-parametric Wilcoxon rank-sum tests and Spearman's rank correlation tests were used. For multiple comparisons, p values were adjusted using the Benjamini-Hochberg correction. A p value threshold of <0.05 was considered statistically significant.

RESOURCE AVAILABILITY

Lead contact

Further information and requests for resources and reagents should be directed to and will be fulfilled by the lead contact, Hiroaki Tateno (h-tateno@aist.go.jp).

Materials availability

This study did not generate new unique reagents.

Data and code availability

The scRNA-seq data have been deposited in Gene Expression Omnibus under accession number GSE304173 and are publicly available as of the date of publication. Raw data and images are available upon request to the corresponding author.

ACKNOWLEDGMENTS

We thank Ms. Keiko Hiemori and Jinko Murakami at National Institute of Advanced Industrial Science and Technology (AIST) for their technical assistance in preparing DNA-barcoded lectins. This work was supported by JSPS KAKEN(22K15660) awarded for HO, JSPS KAKEN(23K26872, 23H04796), and JST A-step (JPMJTR23U6) awarded for HT.

AUTHOR CONTRIBUTIONS

H.O. designed the study, collected and assembled the data, performed the data analysis and interpretation, and wrote the manuscript. H.T. conceived and designed the study, assembled the data, carried out the data analysis and interpretation, and wrote the manuscript.

DECLARATION OF INTERESTS

This paper was applied for a patent in Japan. The application number is "2024–015818."

SUPPLEMENTAL INFORMATION

Supplemental information can be found online at https://doi.org/10.1016/j.stemcr.2025.102631.

Received: November 27, 2024
Revised: August 8, 2025
Accepted: August 8, 2025
Published: September 4, 2025

REFERENCES

Aboul-Soud, M.A.M., Alzahrani, A.J., and Mahmoud, A. (2021). Induced Pluripotent Stem Cells (iPSCs)-Roles in Regenerative Therapies, Disease Modelling and Drug Screening. Cells *10*, 2319. https://doi.org/10.3390/cells10092319.

Alghazali, R., Nugud, A., and El-Serafi, A. (2024). Glycan Modifications as Regulators of Stem Cell Fate. Biology *13*, 76. https://doi.org/10.3390/biology13020076.

Cao, J., Spielmann, M., Qiu, X., Huang, X., Ibrahim, D.M., Hill, A.J., Zhang, F., Mundlos, S., Christiansen, L., Steemers, F.J., et al. (2019). The single-cell transcriptional landscape of mammalian organogenesis. Nature *566*, 496–502. https://doi.org/10.1038/s41586-019-0969-x.

Čaval, T., Alisson-Silva, F., and Schwarz, F. (2023). Roles of glycosylation at the cancer cell surface: opportunities for large scale glycoproteomics. Theranostics *13*, 2605–2615. https://doi.org/10.7150/thno.81760.

Colleoni, S., Galli, C., Giannelli, S.G., Armentero, M.T., Blandini, F., Broccoli, V., and Lazzari, G. (2010). Long-term culture and differentiation of CNS precursors derived from anterior human neural rosettes following exposure to ventralizing factors. Exp. Cell Res. *316*, 1148–1158. https://doi.org/10.1016/j.yexcr.2010.02.013.

Curchoe, C.L., Maurer, J., McKeown, S.J., Cattarossi, G., Cimadamore, F., Nilbratt, M., Snyder, E.Y., Bronner-Fraser, M., Terskikh, A.V., and Terskikh, A.V. (2010). Early acquisition of neural crest competence during hESCs neuralization. PLoS One *5*, e13890. https://doi.org/10.1371/journal.pone.0013890.

de Luzy, I.R., Law, K.C.L., Moriarty, N., Hunt, C.P.J., Durnall, J.C., Thompson, L.H., Nagy, A., Parish, C.L., and Parish, C.L. (2021). Human stem cells harboring a suicide gene improve the safety and standardisation of neural transplants in Parkinsonian rats. Nat. Commun. *12*, 3275. https://doi.org/10.1038/s41467-021-23125-9.

Dworkin, L.A., Clausen, H., and Joshi, H.J. (2022). Applying transcriptomics to studyglycosylation at the cell type level. iScience *25*, 104419. https://doi.org/10.1016/j.isci.2022.104419.

Fair, S.R., Julian, D., Hartlaub, A.M., Pusuluri, S.T., Malik, G., Summerfied, T.L., Zhao, G., Hester, A.B., Ackerman, W.E., 4th, Hollingsworth, E.W., et al. (2020). Electrophysiological Maturation of Cerebral Organoids Correlates with Dynamic Morphological and Cellular Development. Stem Cell Rep. *15*, 855–868. https://doi.org/10.1016/j.stemcr.2020.08.017.

Fujitani, N., Furukawa, J.i., Araki, K., Fujioka, T., Takegawa, Y., Piao, J., Nishioka, T., Tamura, T., Nikaido, T., Ito, M., et al. (2013). Total cellular glycomics allows characterizing cells and streamlining the discovery process for cellular biomarkers. Proc. Natl. Acad. Sci. USA *110*, 2105–2110. https://doi.org/10.1073/pnas.1214233110.

Hao, H., Yuan, Y., Ito, A., Eberand, B.M., Tjondro, H., Cielesh, M., Norris, N., Moreno, C.L., Maxwell, J.W.C., Neely, G.G., et al. (2025). FUT10 and FUT11 are protein O-fucosyltransferases that modify protein EMI domains. Nat. Chem. Biol. *21*, 598–610. https://doi.org/10.1038/s41589-024-01815-x.

Hao, Y., Hao, S., Andersen-Nissen, E., Mauck, W.M., Zheng, S., Butler, A., Lee, M.J., Wilk, A.J., Darby, C., Zager, M., et al. (2021). Integrated analysis of multimodal single-cell data. Cell *184*, 3573–3587.e29. https://doi.org/10.1016/j.cell.2021.04.048.

Hirabayashi, J., Tateno, H., Onuma, Y., and Ito, Y. (2015). A Novel Probe as Surface Glycan Marker of Pluripotent Stem Cells: Research Outcomes and Application to Regenerative Medicine. Adv. Healthcare Mater. *4*, 2520–2529. https://doi.org/10.1002/adhm.201400837.

Isoda, M., Sanosaka, T., Tomooka, R., Mabuchi, Y., Shinozaki, M., Andoh-Noda, T., Banno, S., Mizota, N., Yamaguchi, R., Okano, H., et al. (2023). Mesenchymal properties of iPSC-derived neural progenitors that generate undesired grafts after transplantation. Commun. Biol. *6*, 611. https://doi.org/10.1038/s42003-023-04995-9.

Kanda, Y. (2013). Investigation of the freely available easy-to-use software 'EZR' for medical statistics. Bone Marrow Transplant. *48*, 452–458. https://doi.org/10.1038/bmt.2012.244.

Kearney, C.J., Vervoort, S.J., Ramsbottom, K.M., Todorovski, I., Lelliott, E.J., Zethoven, M., Pijpers, L., Martin, B.P., Semple, T., Martelotto, L., et al. (2021). SUGAR-seq enables simultaneous detection of glycans, epitopes, and the transcriptome in single cells. Sci. Adv. *7*, eabe3610. https://doi.org/10.1126/sciadv.abe3610.

Keisham, S., Saito, S., Kowashi, S., and Tateno, H. (2024). Droplet-Based Glycan and RNA Sequencing for Profiling the Distinct Cellular Glyco-States in Single Cells. Small Methods *8*, e2301338. https://doi.org/10.1002/smtd.202301338.

Kumar, A., Torii, T., Ishino, Y., Muraoka, D., Yoshimura, T., Togayachi, A., Narimatsu, H., Ikenaka, K., Hitoshi, S., and Hitoshi, S. (2013). The Lewis X-related α1,3-fucosyltransferase, Fut10, is required for the maintenance of stem cell populations. J. Biol. Chem. *288*, 28859–28868. https://doi.org/10.1074/jbc.M113.469403.

Lee, A.S., Tang, C., Rao, M.S., Weissman, I.L., and Wu, J.C. (2013). Tumorigenicity as a clinical hurdle for pluripotent stem cell therapies. Nat. Med. *19*, 998–1004. https://doi.org/10.1038/nm.3267.

Li, Z., Liu, L., Jiang, S., Li, Q., Feng, C., Du, Q., Zou, D., Xiao, J., Zhang, Z., Ma, L., and Ma, L. (2021). LncExpDB: an expression database of human long non-coding RNAs. Nucleic Acids Res. *49*, D962–D968. https://doi.org/10.1093/nar/gkaa850.

Liu, X., Zhu, Q., Guo, Y., Xiao, Z., Hu, L., and Xu, Q. (2019). LncRNA LINC00689 promotes the growth, metastasis and glycolysis of glioma cells by targeting miR-338-3p/PKM2 axis. Biomed. Pharmacother. *117*, 109069. https://doi.org/10.1016/j.biopha.2019.109069.

Lucena, M.C., Carvalho-Cruz, P., Donadio, J.L., Oliveira, I.A., de Queiroz, R.M., Marinho-Carvalho, M.M., Sola-Penna, M., de Paula, I.F., Gondim, K.C., McComb, M.E., et al. (2016). Epithelial Mesenchymal Transition Induces Aberrant Glycosylation through Hexosamine Biosynthetic Pathway Activation. J. Biol. Chem. *291*, 12917–12929. https://doi.org/10.1074/jbc.M116.729236.

Lui, J.H., Nowakowski, T.J., Pollen, A.A., Javaherian, A., Kriegstein, A.R., and Oldham, M.C. (2014). Radial glia require PDGFD-PDGFRβ signalling in human but not mouse neocortex. Nature *515*, 264–268. https://doi.org/10.1038/nature13973.

Ma, T., McGregor, M., Giron, L., Xie, G., George, A.F., Abdel-Mohsen, M., and Roan, N.R. (2022). Single-cell glycomics analysis by CyTOF-Lec reveals glycan features defining cells differentially susceptible to HIV. eLife *11*, e78870. https://doi.org/10.7554/eLife.78870.

Minoshima, F., Ozaki, H., Odaka, H., and Tateno, H. (2021). Integrated analysis of glycan and RNA in single cells. iScience *24*, 102882. https://doi.org/10.1016/j.isci.2021.102882.

Nagoshi, N., Okano, H., and Nakamura, M. (2020). Regenerative therapy for spinal cord injury using iPSC technology. Inflamm. Regen. *40*, 40. https://doi.org/10.1186/s41232-020-00149-0.

Nori, S., Okada, Y., Nishimura, S., Sasaki, T., Itakura, G., Kobayashi, Y., Renault-Mihara, F., Shimizu, A., Koya, I., Yoshida, R., et al. (2015). Long-term safety issues of iPSC-based cell therapy in a spinal cord injury model: oncogenic transformation with epithelial-mesenchymal transition. Stem Cell Rep. *4*, 360–373. https://doi.org/10.1016/j.stemcr.2015.01.006.

Odaka, H., Ozaki, H., and Tateno, H. (2022). scGR-seq: Integrated analysis of glycan and RNA in single cells. STAR Protoc. *3*, 101179. https://doi.org/10.1016/j.xpro.2022.101179.

Palma-Tortosa, S., Coll-San Martin, B., Kokaia, Z., and Tornero, D. (2021). Neuronal Replacement in Stem Cell Therapy for Stroke: Filling the Gap. Front. Cell Dev. Biol. *9*, 662636. https://doi.org/10.3389/fcell.2021.662636.

Russell, M.R., Penikis, A., Oldridge, D.A., Alvarez-Dominguez, J.R., McDaniel, L., Diamond, M., Padovan, O., Raman, P., Li, Y., Wei, J. S., et al. (2015). CASC15-S Is a Tumor Suppressor lncRNA at the 6p22 Neuroblastoma Susceptibility Locus. Cancer Res. *75*, 3155–3166. https://doi.org/10.1158/0008-5472.Can-14-3613.

Sugai, K., Fukuzawa, R., Shofuda, T., Fukusumi, H., Kawabata, S., Nishiyama, Y., Higuchi, Y., Kawai, K., Isoda, M., Kanematsu, D., et al. (2016). Pathological classification of human iPSC-derived neural stem/progenitor cells towards safety assessment of transplantation therapy for CNS diseases. Mol. Brain *9*, 85. https://doi.org/10.1186/s13041-016-0265-8.

Takahashi, J. (2020). iPS cell-based therapy for Parkinson's disease: A Kyoto trial. Regen. Ther. *13*, 18–22. https://doi.org/10.1016/j.reth.2020.06.002.

Williams, S.E., Noel, M., Lehoux, S., Cetinbas, M., Xavier, R.J., Sadreyev, R.I., Scolnick, E.M., Smoller, J.W., Cummings, R.D., Mealer, R.G., and Mealer, R.G. (2022). Mammalian brain glycoproteins exhibit diminished glycan complexity compared to other tissues. Nat. Commun. *13*, 275. https://doi.org/10.1038/s41467-021-27781-9.

Xin, J., Zhao, Y.H., Zhang, X.Y., and Tian, L.Q. (2020). LncRNA NFIA-AS2 promotes glioma progression through modulating the miR-655-3p/ZFX axis. Hum. Cell *33*, 1273–1280. https://doi.org/10.1007/s13577-020-00408-9.

Yuan, S.H., Martin, J., Elia, J., Flippin, J., Paramban, R.I., Hefferan, M.P., Vidal, J.G., Mu, Y., Killian, R.L., Israel, M.A., et al. (2011). Cell-surface marker signatures for the isolation of neural stem cells, glia and neurons derived from human pluripotent stem cells. PLoS One *6*, e17540. https://doi.org/10.1371/journal.pone.0017540.

Zhi, F., Wang, Q., Xue, L., Shao, N., Wang, R., Deng, D., Wang, S., Xia, X., Yang, Y., and Yang, Y. (2015). The Use of Three Long Non-Coding RNAs as Potential Prognostic Indicators of Astrocytoma. PLoS One *10*, e0135242. https://doi.org/10.1371/journal.pone.0135242.

Stem Cell Reports, Volume *20*

Supplemental Information

Single-cell glycome and transcriptome profiling uncovers the glycan signature of each cell subpopulation of human iPSC-derived neurons

Haruki Odaka and Hiroaki Tateno

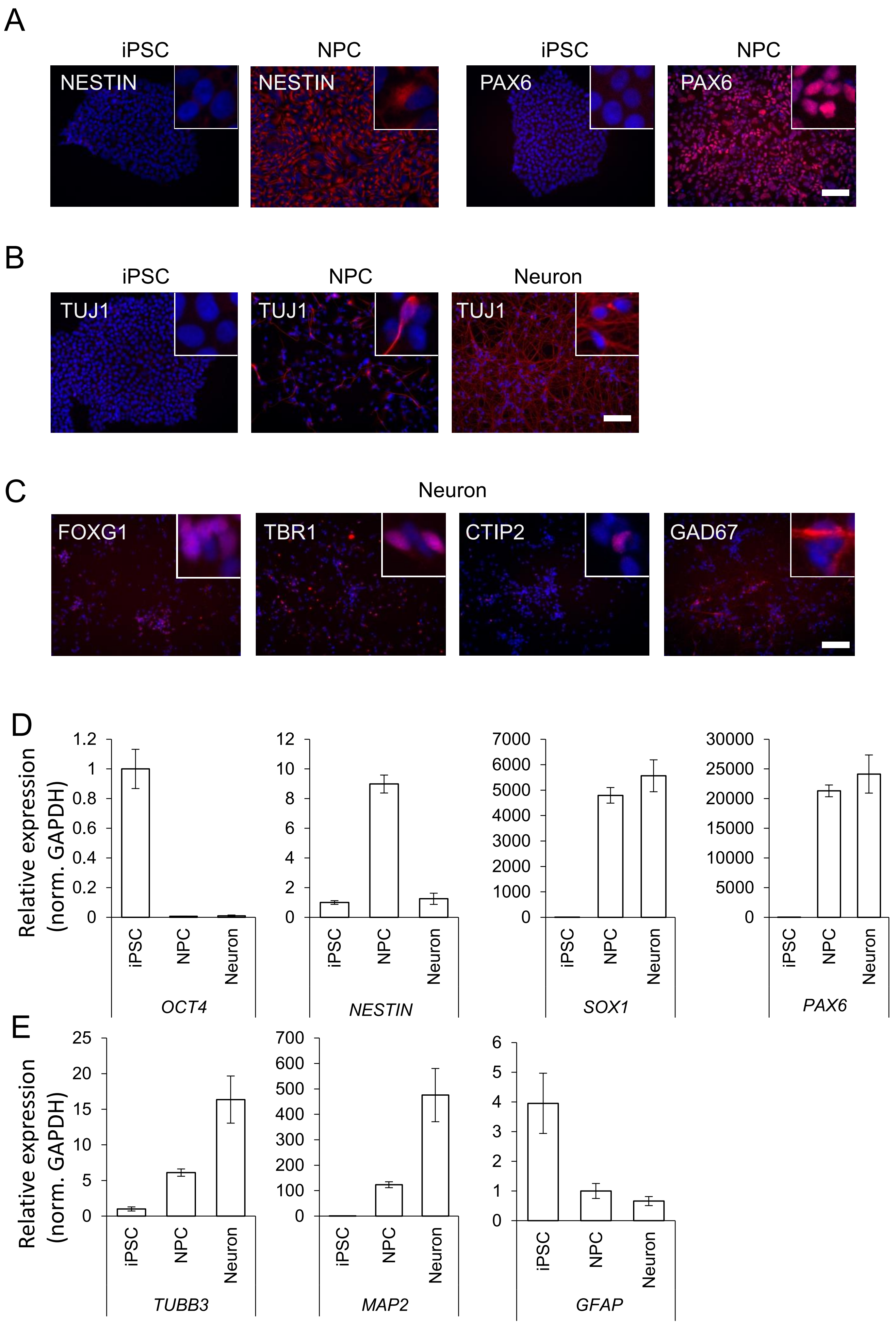

A
iPSC
NPC
iPSC
NPC
NESTIN
NESTIN
PAX6
PAX6
B
iPSC
NPC
Neuron
TUJ1
TUJ1
TUJ1
C
Neuron
FOXG1
TBR1
CTIP2
GAD67
D
Relative expression (norm. GAPDH)
iPSC
NPC
Neuron
OCT4
NESTIN
SOX1
PAX6
E
Relative expression (norm. GAPDH)
TUBB3
MAP2
GFAP

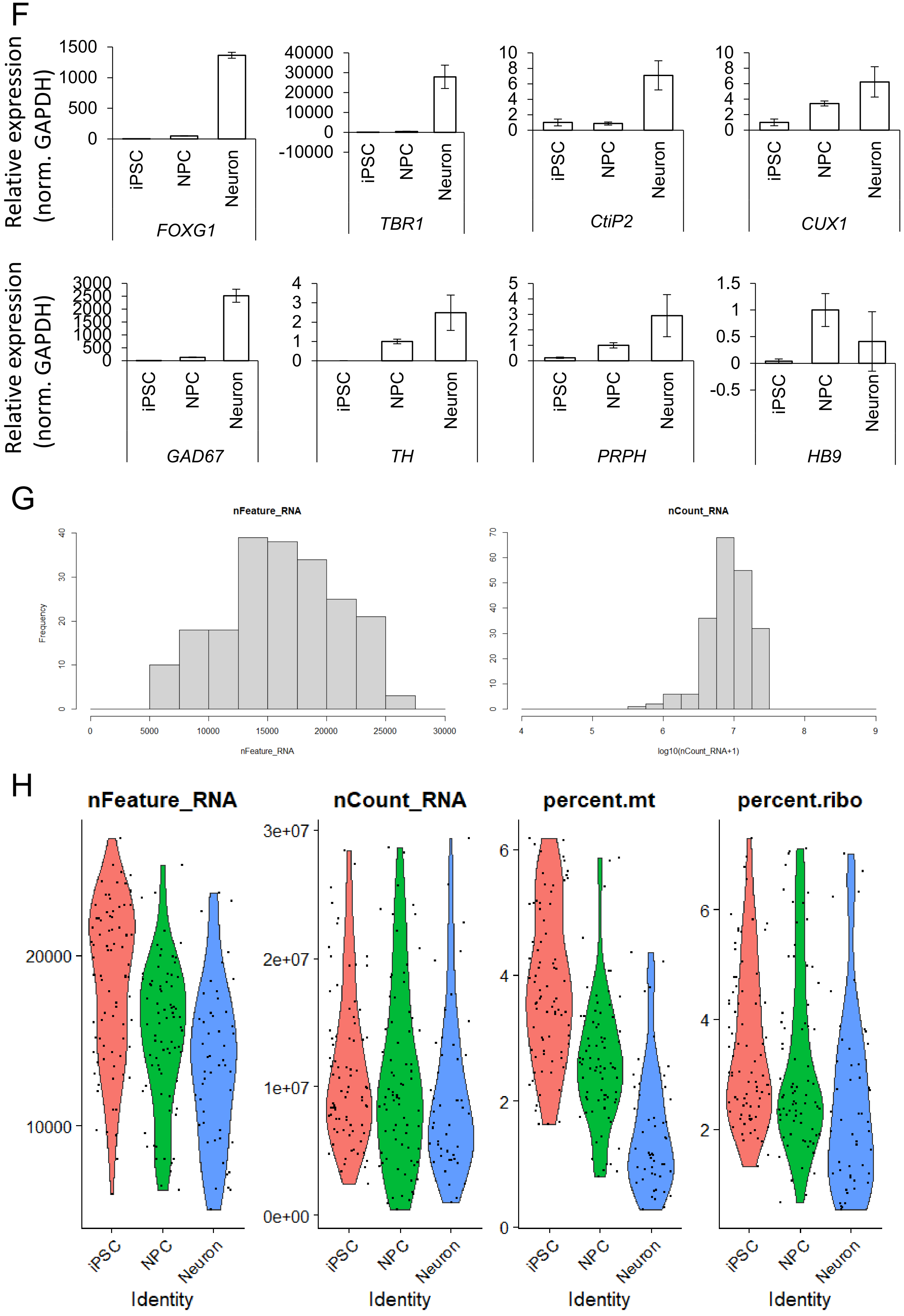
F
Relative expression (norm. GAPDH)
FOXG1
TBR1
CtiP2
CUX1
GAD67
TH
PRPH
HB9
iPSC
NPC
Neuron
G
nFeature_RNA
nCount_RNA
Frequency
log10(nCount_RNA+1)
H
nFeature_RNA
nCount_RNA
percent.mt
percent.ribo
Identity

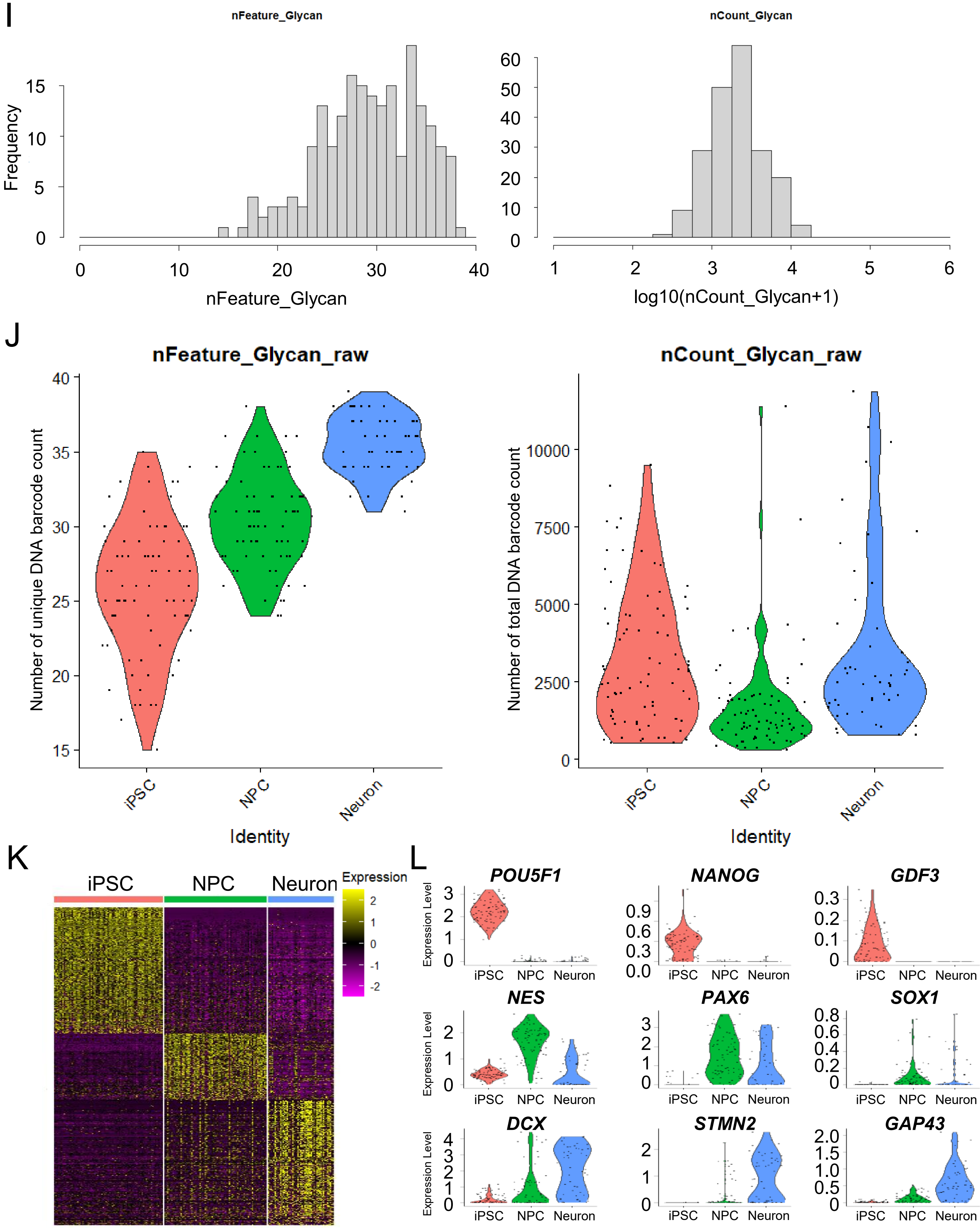

I
nFeature_Glycan
nCount_Glycan
Frequency
0
5
10
15
0
10
20
30
40
nFeature_Glycan
60
50
40
30
20
10
0
1
2
3
4
5
6
log10(nCount_Glycan+1)
J
nFeature_Glycan_raw
nCount_Glycan_raw
Number of unique DNA barcode count
40
35
30
25
20
15
Number of total DNA barcode count
10000
7500
5000
2500
0
iPSC
NPC
Neuron
Identity
K
iPSC
NPC
Neuron
Expression
2
1
0
-1
-2
L
POU5F1
NANOG
GDF3
NES
PAX6
SOX1
DCX
STMN2
GAP43
Expression Level
iPSC
NPC
Neuron

Fig. S1. scGR-seq analysis for iPSC, NPC, and neurons.

(A) Immunofluorescent staining of NPC markers (NESTIN, PAX6) in iPSCs and NPCs. (B) Immunofluorescent staining of a neuron marker (TUJ1) in iPSCs, NPCs and neurons. (C) Immunofluorescent staining of telencepharon (FOXG1), cortical deep-layer neuron (TBR1, CTIP2), and GABAergic neuron (GAD67) markers in neurons. Scale bar: 100 μm (D) Relative mRNA expression of a pluripotent stem cell marker (*OCT4*) and NPC markers (*NESTIN*, *SOX1*, *PAX6*) in iPSCs, NPCs, and neurons (n=3 , each from a separate well). Expression levels of each marker were normalized with *GAPDH* expression. (E) Relative mRNA expression of neuron markers (*TUBB3*, *MAP2*) and an astrocyte marker (*GFAP*) in iPSCs, NPCs, and neurons (n=3 , each from a separate well). (F) Relative mRNA expression of telencepharon (*FOXG1*), cortical deep-layer neuron (*TBR1*, *CTIP2*), cortical upper-layer neuron(*CUX1*), GABAergic neuron (*GAD67*), dopaminergic neuron (*TH*), peripheral neuron (*PRPH*), and motor neuron (*HB9*) markers in iPSCs, NPCs, and neurons (n=3 , each from a separate well). (G) Histogram of the unique genes detected in each cell and the total number of mapped read counts (n=206 cells). (H) Violin plots of the number of unique genes detected in each cell, the total number of mapped read counts, the percentage of mitochondrial genes, and the percentage of genes encoding ribosomal proteins in iPSC (n=81 cells), NPC (n=76 cells), and neurons (n=49 cells). (I) Histogram of the number of unique DNA barcodes (nFeature_Glycan) detected and the total DNA barcode counts (nCount_Glycan) (n=206 cells). (J) Violin plot of number of unique DNA and the total DNA barcode counts in iPSC (n=81 cells), NPC (n=76 cells), and neurons (n=49 cells). (K) Heatmap of differentially expressed genes (DEGs) in iPSC, NPC, and neurons. Criteria for DEG selection was set at log2(FoldChange) > 0.25 and Benjamini-Hochberg adjusted $p < 0.05$ (Mann-Whitney U test). All gene lists are shown in Supplementary Table 1. (L) Violin plots of canonical marker gene expressions of iPSCs (*POU5F1*, *NANOG*, *GDF3*), NPC (*NES*, *PAX6*, *SOX1*) and neurons (*DCX*, *STMN2*, *GAP43*) in each group. iPSC: n=81 cells, NPC: n=76 cells, neuron: n=49 cells.

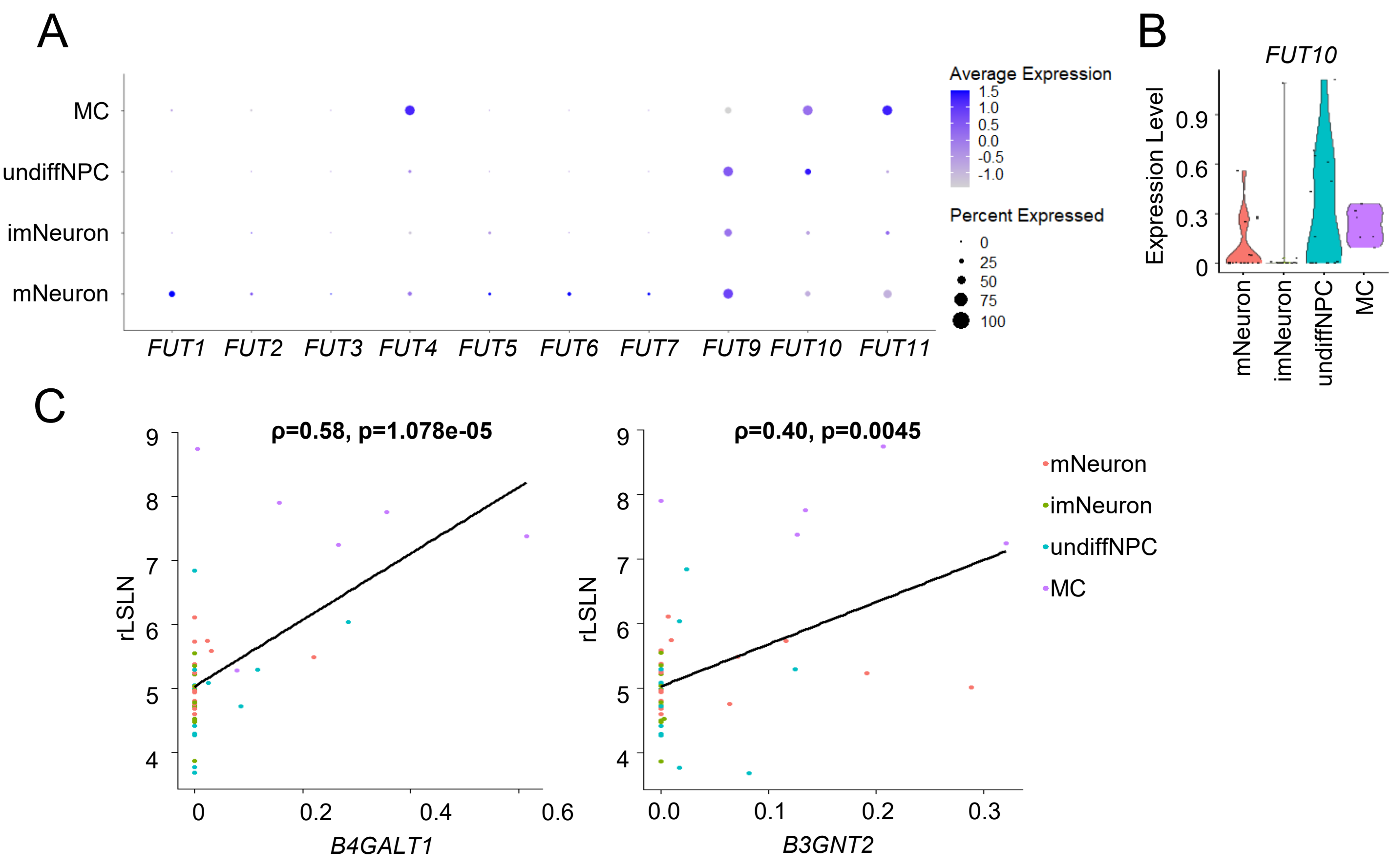

Fig. S2 Gene expression of glycosyltransferases in sub-population of neuron culture.

(A) Dotplots of fucosyltransferase-encoding genes expression in a subpopulation of neurons. (B) Violin plots of the mRNA expression of *FUT10*. (C) Scatter plots showing the relationship between rLSLN binding intensity and the expression levels of *B4GALT1* and *B3GNT2*. The black line represents the fitted regression line. ρ: Spearman's rank correlation coefficient; p: p-value from Spearman's rank correlation test.

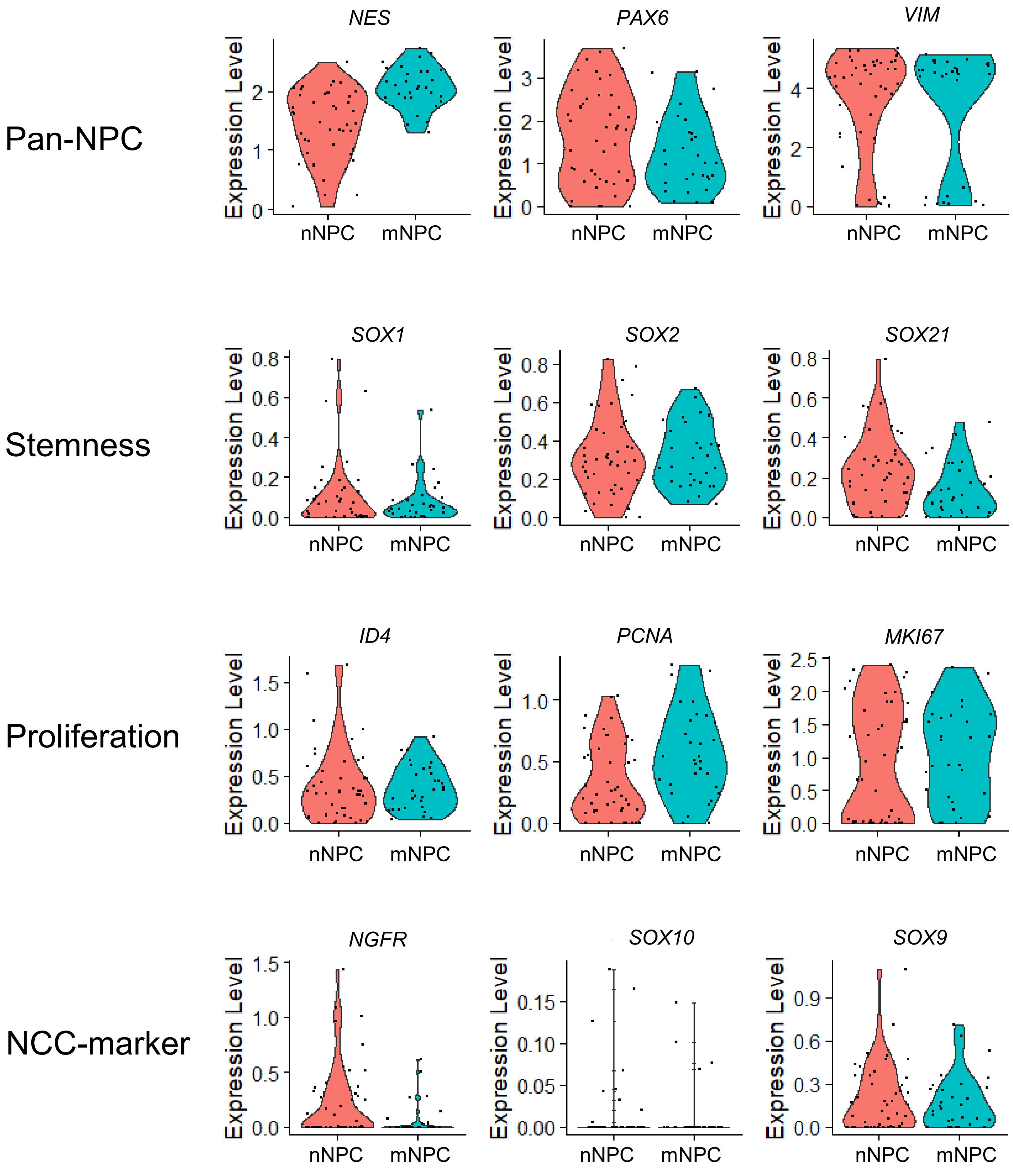

Fig. S3. Gene expression analysis in NPC subpopulations.
Violin plots of the gene expression of stemness markers (SOX1, SOX2 and SOX21), proliferation markers (ID4, PCNA, and MKI67), and neural crest cell markers (NGFR, SOX10, and SOX9) obtained by scRNA-seq analysis.

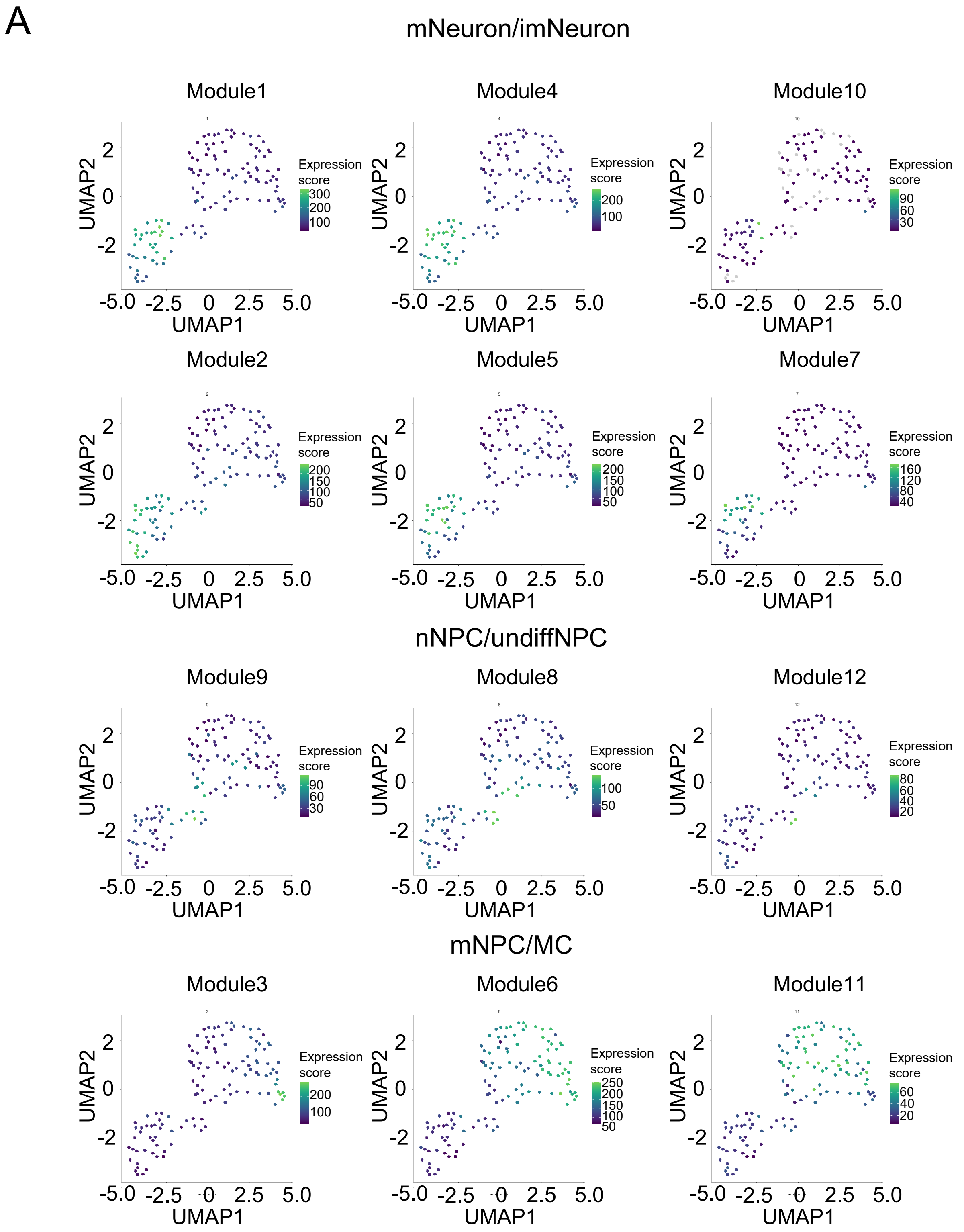
A
mNeuron/imNeuron
Module1
Module4
Module10
Module2
Module5
Module7
nNPC/undiffNPC
Module9
Module8
Module12
mNPC/MC
Module3
Module6
Module11
UMAP1
UMAP2
Expression score

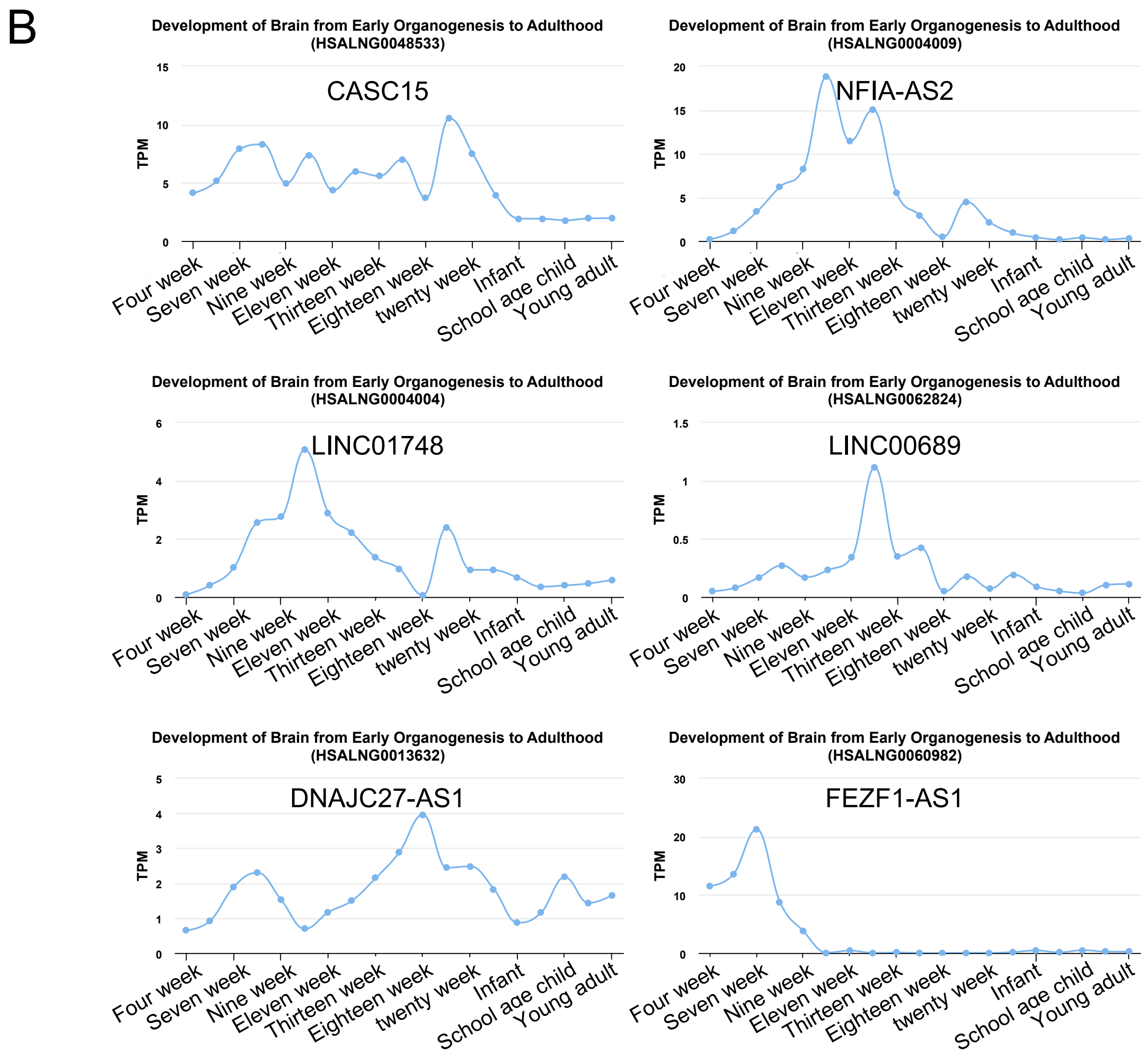
B
Development of Brain from Early Organogenesis to Adulthood (HSALNG0048533)
CASC15
Development of Brain from Early Organogenesis to Adulthood (HSALNG0004009)
NFIA-AS2
Development of Brain from Early Organogenesis to Adulthood (HSALNG0004004)
LINC01748
Development of Brain from Early Organogenesis to Adulthood (HSALNG0062824)
LINC00689
Development of Brain from Early Organogenesis to Adulthood (HSALNG0013632)
DNAJC27-AS1
Development of Brain from Early Organogenesis to Adulthood (HSALNG0060982)
FEZF1-AS1
TPM
Four week
Seven week
Nine week
Eleven week
Thirteen week
Eighteen week
twenty week
Infant
School age child
Young adult

C

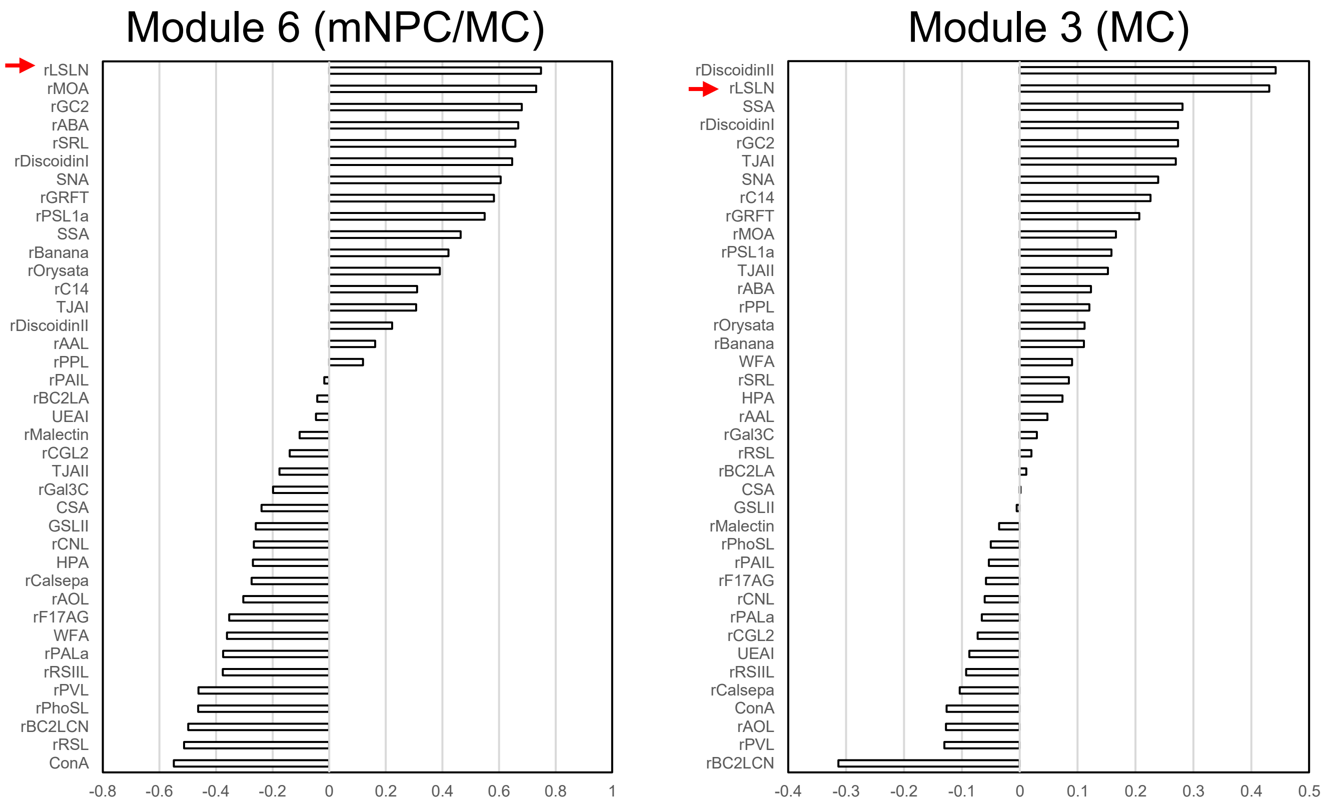
Module 6 (mNPC/MC)
rLSLN
rMOA
rGC2
rABA
rSRL
rDiscoidinI
SNA
rGRFT
rPSL1a
SSA
rBanana
rOrysata
rC14
TJAI
rDiscoidinII
rAAL
rPPL
rPAIL
rBC2LA
UEAI
rMalectin
rCGL2
TJAII
rGal3C
CSA
GSLII
rCNL
HPA
rCalsepa
rAOL
rF17AG
WFA
rPALa
rRSIIL
rPVL
rPhoSL
rBC2LCN
rRSL
ConA
-0.8 -0.6 -0.4 -0.2 0 0.2 0.4 0.6 0.8 1
Module 3 (MC)
rDiscoidinII
rLSLN
SSA
rDiscoidinI
rGC2
TJAI
SNA
rC14
rGRFT
rMOA
rPSL1a
TJAII
rABA
rPPL
rOrysata
rBanana
WFA
rSRL
HPA
rAAL
rGal3C
rRSL
rBC2LA
CSA
GSLII
rMalectin
rPhoSL
rPAIL
rF17AG
rCNL
rPALa
rCGL2
UEAI
rRSIIL
rCalsepa
ConA
rAOL
rPVL
rBC2LCN
-0.4 -0.3 -0.2 -0.1 0 0.1 0.2 0.3 0.4 0.5

D

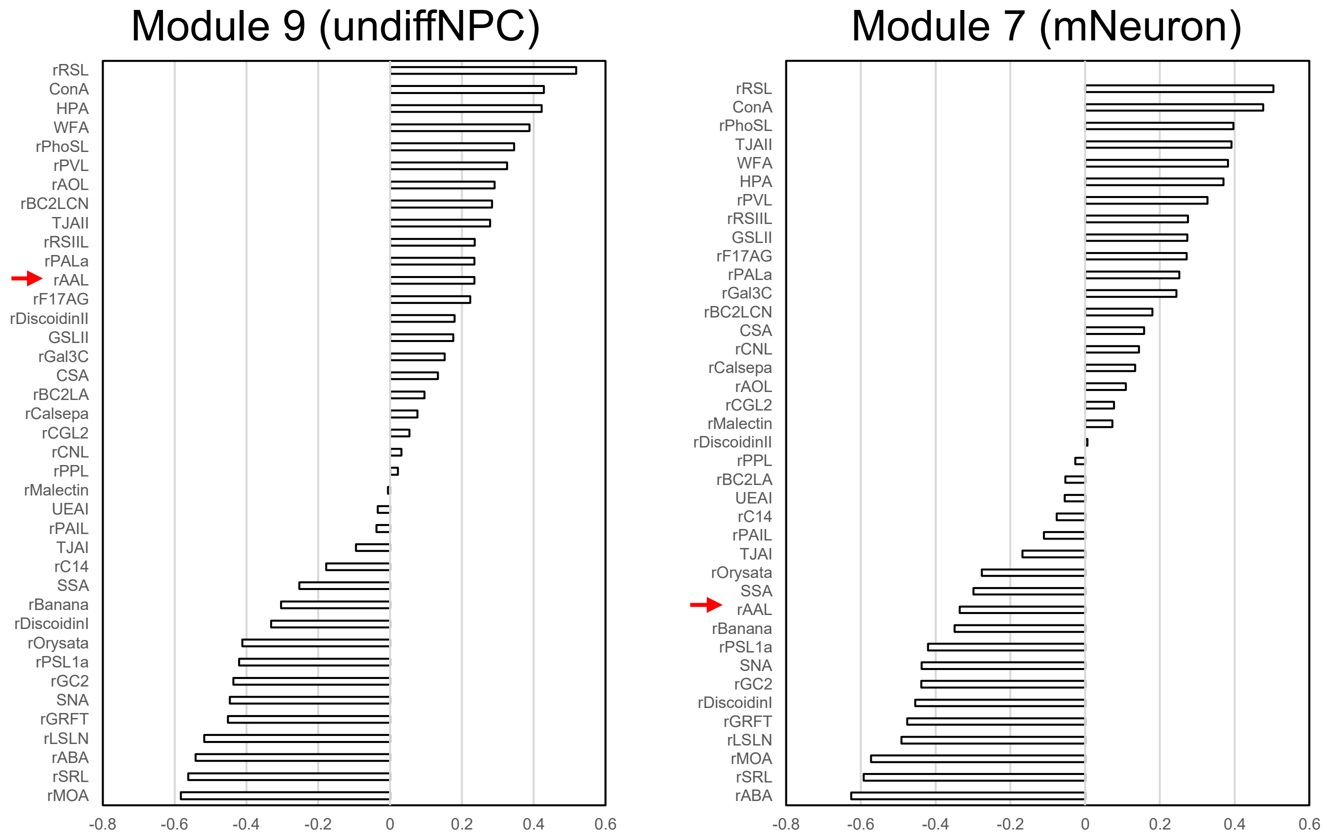
Module 9 (undiffNPC)
rRSL
ConA
HPA
WFA
rPhoSL
rPVL
rAOL
rBC2LCN
TJAII
rRSIIL
rPALa
rAAL
rF17AG
rDiscoidinII
GSLII
rGal3C
CSA
rBC2LA
rCalsepa
rCGL2
rCNL
rPPL
rMalectin
UEAI
rPAIL
TJAI
rC14
SSA
rBanana
rDiscoidinI
rOrysata
rPSL1a
rGC2
SNA
rGRFT
rLSLN
rABA
rSRL
rMOA
-0.8 -0.6 -0.4 -0.2 0 0.2 0.4 0.6
Module 7 (mNeuron)
rRSL
ConA
rPhoSL
TJAII
WFA
HPA
rPVL
rRSIIL
GSLII
rF17AG
rPALa
rGal3C
rBC2LCN
CSA
rCNL
rCalsepa
rAOL
rCGL2
rMalectin
rDiscoidinII
rPPL
rBC2LA
UEAI
rC14
rPAIL
TJAI
rOrysata
SSA
rAAL
rBanana
rPSL1a
SNA
rGC2
rDiscoidinI
rGRFT
rLSLN
rMOA
rSRL
rABA
-0.8 -0.6 -0.4 -0.2 0 0.2 0.4 0.6

E

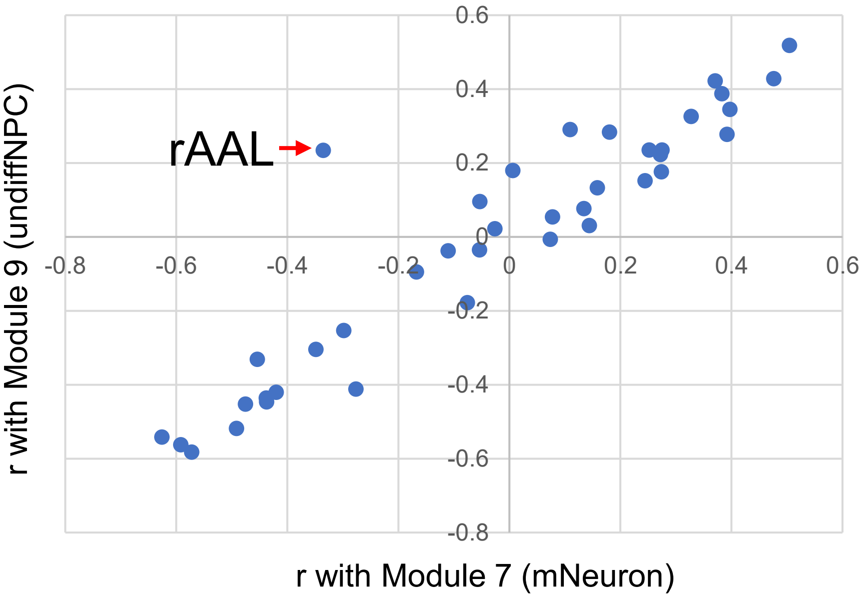

Fig. S4. Co-regulated gene module analysis.

(A) Co-regulated gene module expression plotted on UMAP coordinates for enriched subpopulations. (B) Expression data of long non-coding RNAs within Gene Module 9 were shown from the human lncRNA expression database (LncExpDB). (C) Correlation of mNPC and/or MC-enriched gene modules and lectins. The red arrow indicates rLSLN, which is highly expressed in mNPC and MC. (D) Correlation of undiffNPC or mNeuron-enriched gene modules and lectins. The red arrow indicates rAAL, which showed high binding to undiffNPC. (E) A scatterplot of Pearson correlation coefficients for each lectin with Module7 or Module9. Modules 7 and 9 show a similar lectin correlation pattern. However, rAAL stands out with a distinct correlation compared to the other lectins.

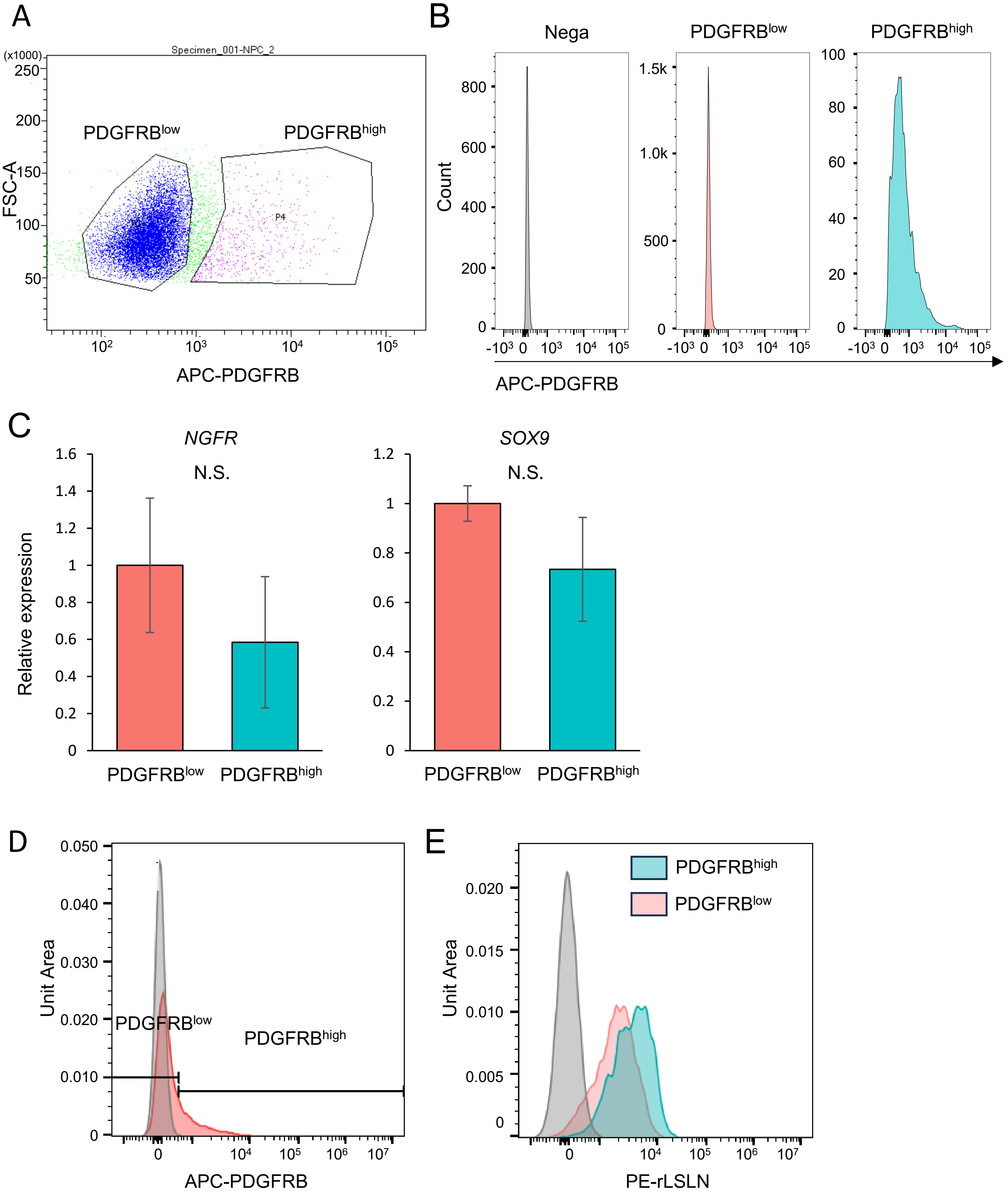
A
(x1000)
Specimen_001-NPC_2
250
200
150
100
50
FSC-A
PDGFRB^low
PDGFRB^high
P4
10^2
10^3
10^4
10^5
APC-PDGFRB
B
Nega
PDGFRB^low
PDGFRB^high
Count
800
600
400
200
0
1.5k
1.0k
500
0
100
80
60
40
20
0
-10^3 0 10^3 10^4 10^5
APC-PDGFRB
C
NGFR
SOX9
N.S.
N.S.
Relative expression
1.6
1.4
1.2
1
0.8
0.6
0.4
0.2
0
1.2
1
0.8
0.6
0.4
0.2
0
PDGFRB^low
PDGFRB^high
D
Unit Area
0.050
0.040
0.030
0.020
0.010
0
PDGFRB^low
PDGFRB^high
0
10^4
10^5
10^6
10^7
APC-PDGFRB
E
PDGFRB^high
PDGFRB^low
Unit Area
0.020
0.015
0.010
0.005
0
0
10^4
10^5
10^6
10^7
PE-rLSLN

Fig. S5. Characterization of PDGFRB^{high} subpopulations.
(A) Fluorescence intensity of APC-conjugated PDGFRB antibody in NPCs and gating of PDGFRB^{low} and PDGFRB^{high} subpopulations for sorting. (B) Fluorescence intensity of APC-conjugated PDGFRB antibody in NPC subpopulations after sorting. (C) Quantitative PCR analysis of NCC marker gene expression (NGFR, SOX9) in PDGFRB^{low} and PDGFRB^{high} NPC subpopulations (n=3, each from an independent sorting experiment). mRNA expression levels were normalized to GAPDH. N.S., not significant. (D) Flow cytometric analysis of APC-conjugated anti-PDGFRB antibody in neural cultures. PDGFRB^{low} and PDGFRB^{high} subpopulations were defined based on fluorescence intensity. (E) Fluorescence intensity of PE-conjugated rLSLN in PDGFRB^{low} and PDGFRB^{high} subpopulations in neural cultures. The gray histogram indicates the unstained (negative control) sample.

Supplemental Methods

Neuralization of iPSCs

201B7 hiPSCs were obtained from RIKEN Bio Resource Center and maintained in mTeSR Plus medium (VERITAS, ST-100-0276) on Matrigel (Corning, 356234) coated plates. Negative mycoplasma and normal chromosomes (46, XX[20]) have been confirmed by suppliers at passage 30. For passage, Subconfluent iPSC colonies were detached by EDTA/PBS (Thermo Fisher, 11665100), dispersed by pipetting and seeded onto Matrigel-coated plates at a ratio of 1:5-1:7. iPSCs were cryopreserved by FreSR™-S (VERITAS) according to the manufacturer's protocol. All experiments were performed with passage 40-60 hiPSCs. Differentiation into NPCs was performed using STEMdiff SMADi Neural Induction Kit according to the manufacturer's protocol (VERITAS). NPCs were expanded until P4 in STEMdiff SMADi medium.
For differentiation into neurons, the NPCs were detached and dissociated into a single cell with Accutase (Innovative Cell Technologie) and seeded on polyethyleneimine (0.07%, Sigma-Aldrich) and laminin (3.3 µg/ml, FUJIFILM Wako)-coated plates. Cells were cultured with neurobasal medium (Thermo Fisher) supplemented with 2% B27 supplement (Thermo Fisher), 1% GlutaMax (Thermo Fisher), and 5 µM DAPT (Sigma-Aldrich) for 1 week. At day 7, the medium was changed to neurobasal plus medium (Thermo Fisher) supplemented with 2% B27 plus supplement (Thermo Fisher) and 1% GlutaMax. Half of the medium was changed twice a week until days 21-28.

Processing of scGR-seq data

Preprocessing of sc-RNAseq data was performed by Subio Platform (version 1.24.5849, Subio inc.) which uses fastp (version 0.22.0) for quality control of FASTQ and HISAT2 (version 2.2.0) for mapping to the human reference genome (GRCh38), and StringTie (version 2.1.1) for calculation of the raw count data. Calculation of raw count data from the FASTQ file of glycan-seq was performed by our previously developed software, Barcode DNA counting system (https://github.com/bioinfo-tsukuba/barcode-dna-counting-system) (Odaka et al., 2022).
The following data processing was performed using R (version 4.0.5). Raw count data of glycan-seq were processed with the TCC R package for normalization of glycan-seq data (Sun, Nishiyama, Shimizu, & Kadota, 2013). Both raw count and TCC-normalized data were imported into Seurat R package (version 4.0.2). To remove the low-quality cells, we set the following quality criteria, number of detected genes > 5000, number of detected genes < 30000, total number of gene count < 30000000, percentage of mitochondria DNA < 7, percentage of ribosomal genes < 7.5, and total number of lectin count < 12500. RNA data was processed using the "NormalizeData" function with the default parameters, the "FindVariableFeatures" function with the parameter selection.method = "vst", nfeatures = 2000", and "ScaleData" function with the default parameters. TCC-normalized glycan data was processed using the "FindVariableFeatures" function with the parameter "selection.method = "vst", nfeatures = 39" , and "ScaleData" function with the parameter "do.scale = FALSE, do.center = TRUE". Principal component analysis (PCA) was then performed on the RNA data and the glycan data using the "RunPCA" function with the default parameters. UMAP plot for RNA data or glycan data was calculated using the "RunUMAP" function with the parameter "dims = 1:20". For the integration of RNA and glycan data, WNN analysis was performed using "FindMultiModalNeighbors" function with the parameter "dims.list =list(1:20, 1:20), k.nn = 10, knn.range = 50", followed by "RunUMAP" function with the default parameter. Differentially expressed genes or lectins were calculated with "FindMarkers" function with the parameter "only.pos = TRUE, logfc.threshold=1" or "logfc.threshold=0", respectively. Adjusted p value for multiple comparisons was calculated using "p.adjust" function with the parameter "BH". GO enrichment analysis was performed using the DAVID 2021 (https://david.ncifcrf.gov/tools.jsp) (Huang da, Sherman, & Lempicki, 2009; Sherman et al., 2022).
For sub-clustering analysis, data from neuron or NPC samples was extracted and data processed until UMAP plot calculation was performed as described above. Clustering analysis of neurons and NPCs was then performed using the "FindClusters" function with the parameter "algorithm = 3, resolution = 1.5, verbose = FALSE" and "algorithm = 3, resolution = 0.5, verbose = FALSE", respectively. Differentially expressed genes or lectins were calculated again with the "FindMarkers" function with the parameter "only.pos =

TRUE" or "logfc.threshold=0", respectively.
For pseudotime analysis, the data from NPCs and neurons were combined and recalculated UMAP plot using "FindMultiModalNeighbors" function with the parameter "dims.list = list(1:3, 1:14), k.nn = 10, knn.range = 10", followed by "RunUMAP" function with the default parameter. The resulting UMAP plot was transfer to the "Monocle3" R package (version 1.0.1) using "as.cell_data_set" function of "SeuratWrappers" R package (version 0.3.0). Pseudotime analysis was performed using "cluster_cells" function with the default parameter, the "learn_graph" function with the parameter "use_partition = TRUE", "order_cells" function with the default parameter. Gene module was determined using the "find_gene_modules" function with the parameter "resolution=3e-2".

Quantitative analysis of fluorescence signal co-localization

To quantify the co-localization of fluorescent signals, we performed co-localization analysis using the Coloc2 plugin in Fiji (ImageJ, version 1.54p) following standard protocols. First, raw fluorescence images were converted to 8-bit grayscale and background noise was removed using the "Subtract Background" function with a rolling ball radius of 200 pixels. Subsequently, co-localization was assessed using Coloc2 with default settings. Pearson's correlation coefficient (r) and Costes' significance test (P-value) were used as primary quantitative indicators of co-localization.

Reference

Odaka, H., Ozaki, H., & Tateno, H. (2022). scGR-seq: Integrated analysis of glycan and RNA in single cells. STAR Protoc, 3(1), 101179. doi:10.1016/j.xpro.2022.101179
Huang da, W., Sherman, B. T., & Lempicki, R. A. (2009). Systematic and integrative analysis of large gene lists using DAVID bioinformatics resources. Nat Protoc, 4(1), 44-57. doi:10.1038/nprot.2008.211
Sherman, B. T., Hao, M., Qiu, J., Jiao, X., Baseler, M. W., Lane, H. C., . . . Chang, W. (2022). DAVID: a web server for functional enrichment analysis and functional annotation of gene lists (2021 update). Nucleic Acids Res, 50(W1), W216-221. doi:10.1093/nar/gkac194
Sun, J., Nishiyama, T., Shimizu, K., & Kadota, K. (2013). TCC: an R package for comparing tag count data with robust normalization strategies. BMC Bioinformatics, 14, 219. doi:10.1186/1471-2105-14-219
